# Changes in healthcare use and cost associated with referrals to social prescribing link workers in England: a matched case–control cohort study

**DOI:** 10.1016/j.eclinm.2026.104102

**Published:** 2026-08-06

**Authors:** Anna Wilding, Luke Munford, Matt Sutton, Morgan Beeson, John Wildman, Stewart W. Mercer, Efundem Agboraw, Paul Wilson

**Affiliations:** a Health Organisation, Policy and Economics, University of Manchester, Manchester, UK; b Centre for Health Economics, Monash University, Melbourne, Australia; c Newcastle University Business School, Newcastle University, Newcastle upon Tyne, UK; d Frederick Douglass Centre, Newcastle University, Newcastle upon Tyne, UK; e Centre for Population Health Studies, Usher Institute, College of Medicine and Veterinary Medicine, University of Edinburgh, Edinburgh, UK

**Keywords:** Social prescribing, Healthcare use, Primary care, Secondary care, Healthcare costs

## Abstract

**Background:**

Social prescribing primarily aims to address patients’ non-clinical needs and increase the appropriateness of healthcare use. A major new national programme in England, introduced in 2019, has led to substantial increases in referrals to social prescribing link workers. Impacts on healthcare use and cost have not been examined. We aimed to address this.

**Methods:**

This case–control cohort study used anonymised electronic healthcare records from the Clinical Practice Research Datalink (CPRD) Aurum dataset, which contains around 25% of the English population to examine healthcare use and costs up to 1 year post-referral. Calliper matching was used to identify five controls for each case based on age and sex, with one control patient selected within the same practice. We matched 168,699 patients with a referral to social prescribing between July 2019 and April 2022 to 1,302,443 non-referred patients. We used multivariable linear regression models to estimate effects on subsequent healthcare use and costs at three timepoints before referral (2 years, 1 year, 6 months) and two timepoints after referral (6 months, 1 year).

**Findings:**

Patients referred to social prescribing had 2.252 more contacts (95% Confidence Interval (CI): 2.089–2.414, 24% relative to mean) with primary and/or secondary care compared to non-referred patients in the first 6 months. This was likely driven by primary care and elective outpatient appointments. By 1 year, this increase was smaller (+0.306, 95% CI: 0.147–0.464) and referred patients had 0.048 (95% CI: −0.084 to −0.012) fewer appointments with general practitioners, compared to non-referred patients. Healthcare costs were <0.1% higher for referred patients (clinically insignificant), due to costs of additional secondary care admissions over both time periods.

**Interpretation:**

The initial increase in use of elective healthcare suggests social prescribing may encourage health-seeking behaviour. The reduction in general practitioner appointments within 1 year suggests social prescribing may result in a shift toward more suitable forms of care. Better integration between social prescribing data platforms and electronic healthcare records would enable future research to estimate the effects of uptake, fidelity and completion of social prescribing link worker referrals on healthcare use and costs.

**Funding:**

10.13039/501100000272National Institute for Health and Care Research.

**Research in context**

**Evidence before this study**

We systematically searched PubMed, PsycINFO, Cochrane Library, Web of Science, and OpenGrey for studies (including grey literature) published in English between Jan 1, 1980, and March 31, 2025, using the search terms “social prescribing”, “community referral”, “healthcare use” and “healthcare cost”. Systematic reviews have identified gaps in knowledge about referrals and healthcare use, particularly noting the dearth of large-scale studies using robust methods. Past evidence of effects of referrals to social prescribing on healthcare utilisation and cost is limited to small pilot studies and before-and-after analyses, and pre-dates the national rollout of social prescribing in England. The 2019 NHS Long Term Plan led to a rapid workforce expansion of social prescribing link workers and subsequent referrals; however, the evidence linking this with healthcare use and cost has yet to be assessed.

**Added value of this study**

This study uses linked electronic primary and secondary care data from Clinical Practice Research Datalink and Hospital Episode Statistics to provide the first national analysis of the effect of referrals to social prescribing on healthcare utilisation and associated costs since the national rollout of link workers in England. We do this in a sample of 168,434 patients with a referral to social prescribing between 1 July 2019 and 31 March 2022, matched to 1,310,723 non-referred patients. We find an initial increase in the use of elective hospital services, followed by a reduction in general practitioner appointments in the longer-term, with no overall difference in use of primary and secondary care. The increases in costs of care were small and not clinically significant.

**Implications of all the available evidence**

Social prescribing aims to address patients’ non-clinical needs to reduce demand for formal healthcare services. Our results suggest the scheme achieves its aims, with an initial increase in use of elective hospital care to address unmet needs and a decrease in general practitioner appointments in the longer term. There were no significant long term changes in use of other types of healthcare. Whilst this study provides a comprehensive analysis of effects on appointments, future work should consider effects on disease onset and a broader range of healthcare services, plus estimate whether the scheme is cost-effective.

## Introduction

Social prescribing is a non-clinical intervention within a healthcare setting that links patients to services in their local community to support their health and well-being.^1^ In England, there has been a large investment in social prescribing through the National Health Service (NHS). The 2019 Long Term Plan aimed to offer more personalised care^2^ and resulted in a large increase in recruitment of social prescribing link workers^3^ and subsequent referrals to the service.^3^, ^4^, ^5^ Social prescribing is part of NHS England's *universal personalised care* model,^6^ where a personalised approach can benefit the health and well-being of those referred, as well as expected positive impacts on the healthcare system in terms of efficiencies and savings. This is achieved through the mechanism of community-centred approaches, where engagement is hypothesised to lead to improvements in individual care and behaviour change.^7^ However, there is a lack of robust quantitative evidence on the impact of referrals to social prescribing on the healthcare system, including on utilisation and cost.^8^

Within England, social prescribing link workers were included as part of the Additional Roles Reimbursement Scheme (ARRS), the main funding source for primary care workforce expansion. This enabled recruitment of other non-GP roles as well, such as pharmacists, advanced practitioners, and physiotherapists. General practices were encouraged to join Primary Care Networks (PCNs) to access this new funding stream. PCNs are groups of general practices that collaborate to serve a population of 30,000–50,000 patients, and they have autonomy in which roles they can recruit. From July 2019, PCNs were allocated funding to hire social prescribing link workers, and NHS England stipulated that by 2022, every PCN should have at least one link worker. The ARRS cost £1 billion in 2022/23, with social prescribing link workers accounting for 13% of the annual cost.^9^^,^^10^ This social prescribing scheme was aimed at all ages and patients who: have one or more long term conditions, need support with low level mental health issues, are lonely or isolated, and/or have complex social needs which affect their wellbeing. Patients are referred by a healthcare professional but can self-refer. It was specified that the link worker should have six to twelve contacts/appointments with each patient over a three-month period. The activities and services patients can attend are dependent on what is available in their local area.

Based on the social prescribing services that were in place before the 2019 NHS Long Term Plan, we understand how link workers can work to support access and engagement with services that address patient needs.^11^^,^^12^ However, the evidence base for the impact of social prescribing link workers on health outcomes and service utilisation is more limited. Successive systematic reviews have highlighted a literature dominated by small scale, poorly designed studies with a high risk of bias, making it difficult to reliably judge potential impact.^8^^,^^13^ The scale of the NHS England social prescribing scheme offers an opportunity to address this shortcoming and provide robust evaluation of the impact of social prescribing link workers on primary care. In this paper, we aim to estimate whether referrals to social prescribing are associated with changes in healthcare use and cost in secondary and primary care.

## Methods

### Study design and ethics

We used a case–control study design, with calliper matching to identify five controls for each case based on age (± 5 years) and sex. We selected one control patient within the same practice and four control patients from other practices. Cases were indexed at their referral date. Controls were assigned a pseudo-index date drawn at random from the overall distribution of case referral dates. This ensured that cases and controls shared the same distribution of before- and after-periods.

The study used anonymised electronic healthcare records from the Clinical Practice Research Datalink (CPRD) Aurum dataset. CPRD Aurum contains around 25% of the English population^14^^,^^15^ and is broadly representative in terms of age, sex, ethnicity, socioeconomic deprivation and regions of England.^16^ The inclusion criteria for our study were patients registered with their general practice for at least 1 year during the study treatment period (1 April 2017–31 March 2023) and classified by CPRD as acceptable for research use. An additional inclusion criterion was patients who had linkage using their home address to small area data and to their secondary care records through hospital episode statistics (HES).

Within this sample, we matched on combinations of the following predictors of referral: number of long-term conditions; diagnosed anxiety or depression; number of primary care contacts in the 6 months prior to referral; and month and year of referral. We include justification for the inclusion in Supplementary Table S3. We retained all control patients that matched to each case and weighted them by the inverse of their count. In an additional sensitivity analysis, we also matched on number of primary care contacts in the year prior to referral, as well as variables listed in Supplementary Table S3. Since this reduced the number of matched cases by 39.5%, we kept the original matched sample, comprising 95.9% of cases (4.1% are unmatched), as the main analysis. This stricter matching highlights that the sample of referred populations has different primary care use prior to referral than the control group, compared to when we use only 6 months prior.

Regarding ethics review, the study protocol was approved by the Independent Scientific Advisory Committee of the Medicines and Healthcare products Regulatory Agency (23_002643). Full details are available online at https://www.cprd.com/approved-studies/multi-region-evaluation-national-roll-out-social-prescribing-link-workers-primary. The requirement for written informed consent was waived, as CPRD has been granted generic ethics approval, owing to the use of only anonymised data and linked anonymised NHS health-care data.

### Referrals to social prescribing

We used the SNOMED code “*871731000000106–Referral to Social Prescribing”* recorded in the observation files to identify referrals. The NHS created this code to monitor the number of referrals attributed to social prescribing link workers employed through the Additional Roles Reimbursement Scheme.^17^ This created a standardised measure of tracking referrals within records across general practices. We used the first mention of this code between 1 July 2019 and 31 March 2022 to identify patients referred to social prescribing. If patients are re-referred, those interactions are not included.

### Outcomes

We calculated healthcare use and associated costs over the following time periods: 2 years, 1 year and 6 months before referral; and 6 months and 1 year after referral. We only follow patients for 1 year after referral, as HES linkages are only available to 31 March 2023.

We created a combined count of all contacts with primary and secondary care. We also counted contacts of various types as outlined below. We define a contact as any appointment or admission.

For primary care contacts, we extracted appointments from the consultation files for the following staff types: General Practitioner, Nurse, Healthcare Assistant and other roles, which include Pharmacist, Physiotherapist, Physician Associate, Paramedic, Midwife, Counsellor, Phlebotomist, Podiatrist, Therapist, Advanced Practitioner, Radiologist and Dietician. We restricted appointments to face-to-face, telephone and online modes. We followed previous methods used to identify appointments in CPRD^18^ and outlined all the codes and descriptions associated with each staff role and appointment type in Supplementary Code 1. From this, we calculated the number of contacts with all primary care staff and with general practitioners. Contacts were costed using 2022 unit costs^19^ for each role (see Supplementary Table S1).

For secondary care contacts, we counted numbers of outpatient appointments and of elective and emergency hospital admissions, excluding maternity admissions. This includes anything record in HES (secondary care records). We used the 2022–23 national tariff to estimate hospital care costs (see Supplementary Table S2). Costs are expressed in 2022 prices.

### Covariates

We measured number of long-term conditions at the point of referral. We used the Elixhauser list of diagnostic codes.^20^ We used the earliest event date to identify the diagnosis from the observational files. From this, we created a morbidity measure categorising individuals as having no long-term conditions, one long-term condition, two long-term conditions, three long-term conditions, or four or more long-term conditions. We separately identified patients with a common mental health condition diagnosis (anxiety and/or depression), as this is a target group for social prescribing.

In addition to biological sex and age, we extracted ethnicity from the six categories provided by HES linkages: Asian, Black, Mixed, White, Other, or unknown. These were derived from the ONS Ethnicity definition. For socioeconomic deprivation, we used deciles of the Index of Multiple Deprivation 2019—Income Domain. For geographical location, we use government office regions, which indicate if patient resides in one of the following areas: Yorkshire and Humberside, North West, East of England, East Midlands, West Midlands, North East, London, South East and South West.

### Statistical analysis

For referrals to social prescribing, we present descriptive statistics of counts of referrals to social prescribing for each month and year across 1 July 2019–31 March 2022.

For descriptive statistics of patient characteristics and outcomes, we present frequencies and percentages for categorical/binary variables, and for continuous variables, we present means and standard deviations for referred and non-referred populations, which are adjusted using matching weights. We present unweighted values in the Supplementary Tables.

For our regression analysis, we used multivariable linear regression models with lagged dependent variables^21^ to estimate the effect of referrals to social prescribing on patients’ healthcare use and cost after referral. We adjusted for past healthcare use or cost (depending on the outcome) in the 2 years prior to referral, age, including a quadratic term, biological sex, ethnicity, deprivation decile, morbidity count, government office region, month and year fixed effects. Supplementary Table S4 describes the rationale for inclusion of these variables. We used analytical weights calculated from the matching process. In the main analysis, we clustered the standard errors at general practice level. In sensitivity analyses, we used a multi-level model with random effects for general practice at the higher level. For all models, we present the main effect: coefficient on referral to social prescribing. In the Supplementary Files, we show the covariate effect, the unweighted models for the linear models, multivariable regression models and negative binomial models (post-hoc). All analyses were conducted in Stata v18.

### Role of the funding source

The funders had no involvement in study design, data collection, data analyses, data interpretation, or the writing of the report.

## Results

### Volume of social prescribing referrals

**Fig. 1:**
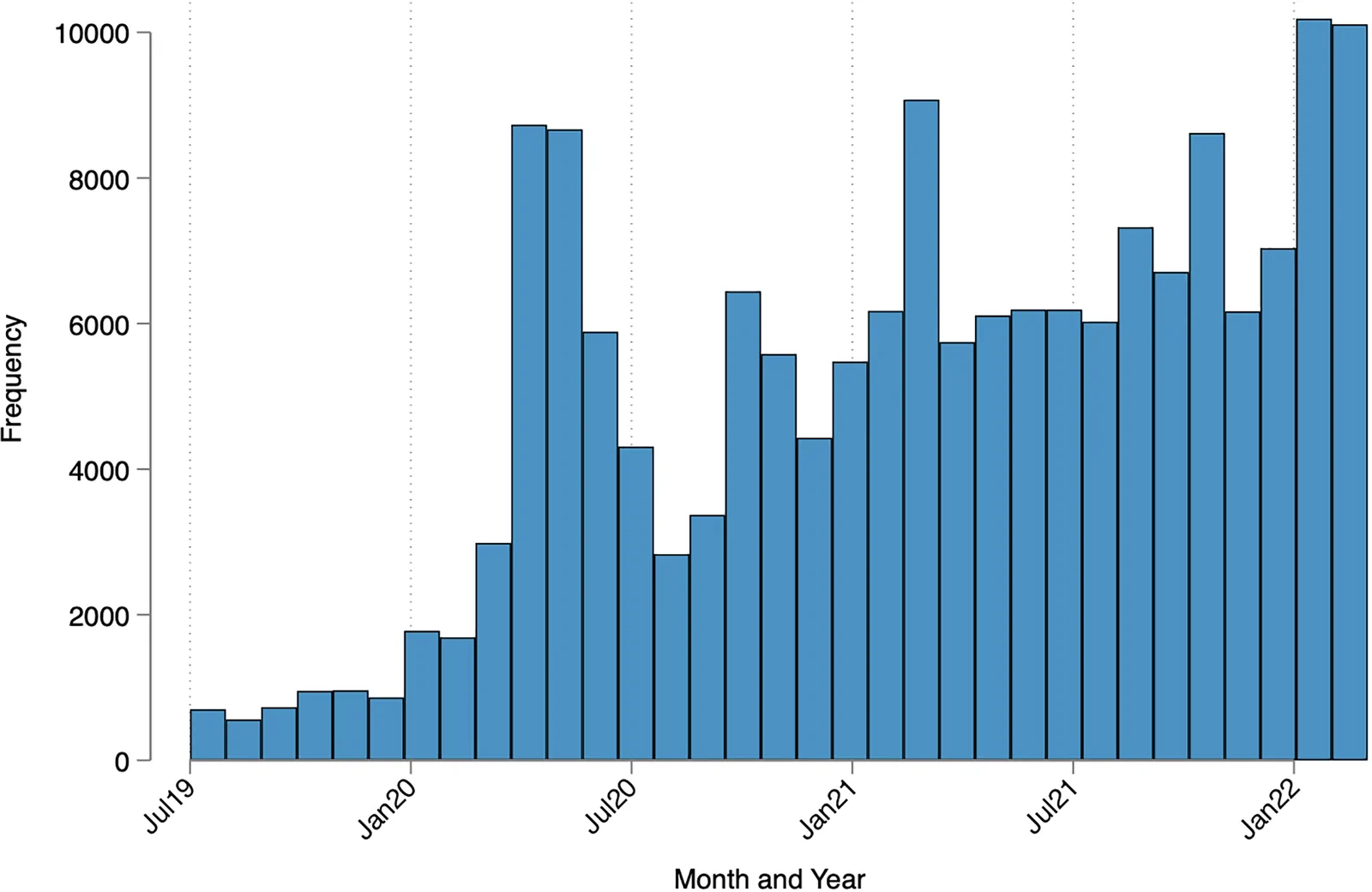
Number of patients referred to social prescribing across the study period (1 July 2019–31 March 2022).

The volume of referrals to social prescribing across our study period (1 July 2019–31 March 2022) is presented in Fig. 1. We show the same graph in Supplementary Figure S1 for our control group, based on their randomly assigned referral dates.

In the early years of the rollout of social prescribing link workers in primary care, the number of referrals was low; this increased from 2020 onwards. There was a higher number of referrals in March 2021, likely due to the payment deadline for the impact and investment fund, a financial incentive for PCNs for referring patients to social prescribing. In 2022, there was a further increase in the volume of referrals.

### Patient characteristics

Table 1 contains descriptive statistics by referral status for the matched populations. We provide the unweighted descriptive statistics in Supplementary Table S5, and for unmatched patients in Supplementary Table S6. The populations are very similar in the characteristics used for matching: anxiety or depression, and the number of long-term conditions. The referred population has a higher proportion of people of Black ethnicity. There is a lower proportion of people from the most deprived deciles in the referred population. There is a much higher proportion of patients from London in the referred population.

**Table 1:** Descriptive statistics of patient characteristics split by referral status (Mean and Standard Deviation, and Percentage and Frequency).

	Referred		Non-referred	
	%/Mean	(SD)/Freq	%/Mean	(SD)/Freq
Patients	168,699		1,302,443	
Age	61.14	(19.67)	62.87	(18.74)
Female	60.2%	101,601	66.9%	871,334
Male	39.8%	67,098	33.1%	431,109
Anxiety or depression	49.0%	82,585	48.8%	635,592
Morbidity measure				
0 LTC	6.7%	11,356	6.7%	87,264
1 LTC	15.3%	25,839	15.3%	199,274
2 LTCs	17.8%	30,079	17.7%	230,532
3 LTCs	16.3%	27,508	16.5%	214,903
4 or more LTCs	43.8%	73,917	43.8%	570,470
Ethnicity				
Asian	7.8%	13,134	6.7%	87,264
Black	7.0%	11,789	3.4%	44,283
Mixed	1.4%	2318	1.0%	13,024
White	82.4%	138,925	87.6%	1,140,940
Other	0.2%	354	0.2%	2605
Unknown	1.3%	2179	1.2%	15,629
Income deprivation decile				
1 = Most	7.4%	12,499	9.7%	126,337
2	8.3%	13,961	10.3%	134,152
3	8.5%	14,298	10.5%	136,757
4	8.4%	14,224	9.8%	127,639
5	8.8%	14,920	9.8%	127,639
6	9.6%	16,275	9.8%	127,639
7	11.5%	19,387	10.4%	135,454
8	12.7%	21,347	10.0%	130,244
9	12.9%	21,770	10.2%	132,849
10 = Least	11.9%	20,018	9.6%	125,035
Region				
North East	1.0%	1691	2.2%	28,654
North West	4.1%	6934	4.1%	53,400
Yorkshire and Humberside	25.2%	42,455	15.6%	203,181
East Midlands	5.1%	8585	3.8%	49,493
West Midlands	16.3%	27,474	18.9%	246,162
East of England	19.5%	32,927	20.7%	269,606
London	10.8%	18,244	13.8%	179,737
South East	15.0%	25,246	17.4%	226,625
South West	3.0%	5143	3.5%	45,586

**Note:** % = Percentage, SD, Standard Deviation; Freq, Frequency; LTC, Long Term Conditions. This cohort was exact matched on the following variables number of long-term conditions; diagnosed anxiety or depression; number of primary care appointments in the 6 months prior to referral; and month and year of referral. Means are adjusted using sampling weights from exact matching.

### Healthcare use and costs

Table 2 provides descriptive statistics on use and total costs of healthcare before and after referral. Supplementary Table S7 provides the same for healthcare costs. The average values of use and cost for the unweighted non-referred population are provided in Supplementary Table S8.

**Table 2:** Healthcare use and costs pre- and post-referral by referral status (Mean and Standard Deviation).

	Time frame	Referred				Non-referred			
		Pre-referral		Post-referral		Pre-referral^a^		Post-referral^a^	
		Mean	(SD)	Mean	(SD)	Mean	(SD)	Mean	(SD)
Secondary and primary care	*6 months*	9.96	(9.21)	10.32	(10.23)	9.62	(8.90)	7.89	(9.00)
Primary care	*6 months*	7.60	(6.41)	7.89	(7.56)	7.53	(6.30)	5.92	(6.40)
General practitioner	*6 months*	4.63	(4.62)	4.50	(5.03)	4.28	(4.40)	3.31	(4.22)
Secondary care	*6 months*	2.36	(5.45)	2.42	(5.61)	2.09	(4.99)	1.98	(4.97)
Outpatients	*6 months*	1.84	(4.32)	1.91	(4.50)	1.66	(4.07)	1.59	(4.00)
Admitted patient care	*6 months*	0.52	(2.48)	0.51	(2.51)	0.43	(2.10)	0.39	(2.16)
Elective	*6 months*	0.30	(2.32)	0.29	(2.35)	0.24	(1.95)	0.23	(2.03)
Emergency	*6 months*	0.21	(0.64)	0.21	(0.66)	0.17	(0.58)	0.14	(0.53)
Secondary and primary care	*1 year*	19.08	(17.46)	17.83	(15.94)	15.48	(15.84)	17.03	(15.46)
Primary care	*1 year*	14.49	(12.69)	13.39	(10.96)	11.63	(11.11)	13.11	(10.82)
General practitioner	*1 year*	8.28	(8.45)	8.15	(7.73)	6.40	(7.17)	7.45	(7.31)
Secondary care	*1 year*	4.59	(9.51)	4.45	(9.35)	3.85	(8.72)	3.92	(8.60)
Outpatients	*1 year*	3.63	(7.41)	3.48	(7.20)	3.09	(6.85)	3.13	(6.91)
Admitted patient care	*1 year*	0.95	(4.59)	0.97	(4.56)	0.77	(4.04)	0.79	(3.79)
Elective	*1 year*	0.55	(4.37)	0.57	(4.34)	0.46	(3.84)	0.45	(3.58)
Emergency	*1 year*	0.38	(1.01)	0.37	(0.97)	0.28	(0.83)	0.31	(0.88)
Costs (£)									
Secondary and primary care	*6 months*	1062	(3168)	1022	(3066)	799	(2669)	891	(2854)
Secondary and primary care	*1 year*	1951	(4772)	1836	(4687)	1560	(4185)	1630	(4343)
Observations		168,699				1,302,443			

Note: SD, Standard Deviation.

a Referral date here is the index date, this is randomised to matched density of referrals across study period.

People referred to social prescribing had, on average, 9.9 contacts with primary and secondary care in the 6 months leading up to their referral and slightly more (10.3) contacts in the 6 months following referral. They had fewer (17.8) contacts in the year following referral compared to the average of 19.1 contacts in the year leading up to referral. In contrast, non-referred patients had fewer contacts in the 6 months after their matched patients’ referral dates than they did in the 6 months before (7.9 versus 9.6 contacts) and more contacts in the year after compared to the year before (17.0 versus 15.4 contacts).

The overall contacts with healthcare are driven by primary care, with referred patients having an average of 7.6 appointments within primary care in the 6 months leading up to referral, with 4.6 of those with a general practitioner, and the remaining with other healthcare professionals. The opposite is true for costs; primary care represents a lower proportion of the overall costs, whereas secondary care represents a higher proportion of overall costs, particularly admitted patient care.

Supplementary Tables S9 and S10 contain the healthcare use and cost for the unmatched patients from our case–control design. Supplementary Tables S11 and S12 contain the healthcare use and cost for the sub-sample where observations were matched on the 1-year prior primary care contacts, in addition to matching variables used for the sample in Table 2 and Supplementary Table S6. These samples are more similar in terms of 1 year post-utilisation. This could also be due to the smaller sample size.

### Regression results

There is an increase of 2.52 (95% Confidence Interval (CI) (2.10–2.43)) contacts in the 6 months after referral for those referred to social prescribing compared to those not referred (Table 3). This was explained by increases in elective care, specifically primary care and outpatient appointments. The increase in overall contacts was much smaller (0.31; 95% CI 0.15–0.46) after 1 year. There is a similar pattern in cost of care, with an increase of £42.42 (95% CI £21.07–£63.77) in the 6 months after referral and a smaller increase of £26.35 (95% CI £6.96–£45.74) after 1 year. These cost differences were explained by admitted patient care costs (columns 6–8). These increases in costs are small in both relative and absolute terms and clinically negligible when compared to the means in Table 2, and Supplementary Table S7.

**Table 3:** Estimated effects of referral to social prescribing on healthcare use and cost at 6 months and 1 year after referral (Coefficient, 95% Confidence Intervals, p-values).

	(1)	(2)	(3)	(4)	(5)	(6)	(7)	(8)
	Primary care and hospital	All primary care	General practitioner	All hospital	Outpatient	Admitted patient care	Admitted patient care–elective	Admitted patient care—emergency
Contacts								
6 months post	2.252∗∗∗	1.961∗∗∗	0.914∗∗∗	0.248∗∗∗	0.196∗∗∗	0.054∗∗∗	0.009	0.055∗∗∗
	(2.089–2.414)	(1.812–2.111)	(0.855–0.972)	(0.199–0.296)	(0.152–0.240)	(0.041–0.066)	(−0.003 to 0.020)	(0.051–0.059)
	<0.001	<0.001	<0.001	<0.001	<0.001	<0.001	0.129	<0.001
1 year post	0.306∗∗∗	0.188∗∗	−0.048∗∗	−0.022	−0.026	0.005	−0.001	0.006∗∗
	(0.147–0.464)	(0.050–0.326)	(−0.084 to −0.012)	(−0.053 to 0.009)	(−0.052 to 0.000)	(−0.005 to 0.015)	(−0.009 to 0.007)	(0.002–0.010)
	<0.001	0.008	0.010	0.172	0.054	0.315	0.775	0.005
Cost (£s)								
6 months post	42.415∗∗∗	0.225	−1.660	40.600∗∗∗	2.426	38.580∗∗∗	3.000	36.909∗∗∗
	(21.065–63.766)	(−2.496 to 2.947)	(−3.485 to 0.166)	(19.251–61.949)	(−0.850 to 5.703)	(18.663–58.497)	(−4.761 to 10.761)	(20.768–53.049)
	<0.001	0.871	0.075	<0.001	0.147	<0.001	0.448	<0.001
1 year post	26.351∗∗	2.881	−2.149∗∗	26.429∗∗	−0.331	27.012∗∗	4.690	24.268∗∗∗
	(6.958–45.744)	(−0.438 to 6.201)	(−3.759 to −0.539)	(7.029–45.828)	(−3.918 to 3.256)	(9.200–44.824)	(−4.336 to 13.716)	(10.483–38.053)
	0.008	0.089	0.009	0.008	0.856	0.003	0.308	0.001

**Note:** N = 1,479,157, 95% Confidence Intervals based on clustered standard errors at general practice (N = 1432) level in parentheses. ∗p < 0.05, ∗∗p < 0.1, ∗∗∗p < 0.001. Models include covariates: outcome 2 years prior, age, ageˆ2, female, ethnicity group, IMD, Income Domain Decile, government office region, and month and year fixed effects of referral date. Models include weights from exact matching. Coefficients from covariates can be found in Supplementary Tables S16–S19.

For primary care, post-referral, there is an increase in appointment use in the short term (6 months) by 1.96 (95% CI (1.81–2.11)). General practitioner appointments are contributing to this, with an increase of 0.91 appointments (95% CI (0.86–0.97)). However, in the longer term (1-year post-referral), there is no significant effect on all primary care appointments but a significant decrease in general practitioner appointments by 0.048 (05% CI (−0.08 to −0.01)). This is likely to be clinically negligible. In terms of costs, there is no significant effect for primary care as a whole, but a significant decrease in general practitioner costs in the long term by £2.15 (95% CI(−£3.76 to −£0.534)). However, this effect is clinically small as it represents as 1.5% decrease in the mean.

Supplementary Table S13 contains the unweighted and unadjusted models, Supplementary Table S14 contains the weighted and unadjusted models (post-hoc), and Supplementary Table S15 contains the unweighted and unadjusted models. Supplementary Tables S16–S19 contain the covariate effects from the Models within Table 3. Supplementary Table S20 contains the results from the stricter matching criteria. From the summary statistics, the pre-periods were more similar than those shown in the main models, particularly the 1-year prior. In terms of regression results, the results are broadly consistent with Table 3. The main changes relate to primary care, with a reduction in magnitude in the 6 months post-referral, and general practitioner appointments not being significant in the 1-year post-referral. Supplementary Table S21 contains the results from the multilevel modelling, where coefficients are consistent with Table 3. Supplementary Table S22 contains the negative binomial models (post-hoc).

## Discussion

Since the rollout of social prescribing link workers across primary care networks in England, there has been a substantial increase in the number of patients referred to social prescribing. We find that referral is associated with 2.52 more healthcare contacts (95% CI (2.09–2.42) in the short term (6 months after referral). In the longer term (1 year after referral), this difference between referred and non-referred patients reduces to just 0.31 additional contacts (95% CI (0.15–0.46). Referral is associated with a reduction in general practitioner appointments within 1 year. In terms of costs, there is an increase in the cost of care in the short term by £42.42 per referred patient (95% CI (£21.07–£63.77)), with this increase decreasing to £26.35 per patient in the longer term (95% CI (£6.96–£45.74)). These costs are driven by secondary care rather than primary care and are small in relative and absolute terms, and clinically insignificant.

Our findings suggest that among 168,699 patients referred to social prescribing between 1 July 2019 and 31 March 2022, there was an initial increase in healthcare utilisation, particularly for primary care and outpatient appointments, followed by a subsequent decrease or no significant difference compared with those not referred. Referrals to social prescribing are associated with an increase in elective care in the short term, but in the longer term, patients exhibit similar patterns of healthcare use to those who were not referred. As the scheme is designed for individuals requiring non-clinical or holistic support at the point of referral, these non-significant differences in the longer term suggest that social prescribing may be effective in enabling patients to address non-medical needs, stabilise their use of healthcare services, and seek support from appropriate community resources rather than continued clinical care.

Past research on social prescribing has evaluated individual services and much of the existing evidence is limited by poor design and reporting.^8^^,^^13^^,^^22^ This study is the first of its kind to have generalisable findings for both healthcare use and cost. Our findings are able to address concerns outlined in the previous systematic review of study design, lack of counterfactuals, short follow-up periods and non-generalisable sample.^8^ We find a downward trend in healthcare utilisation in the longer term as found in previous studies.^23^^,^^24^ However, we do find increased care in the shorter term for both primary and secondary care. This could be due to having a more complete picture of care with linked healthcare records, or from improved model specifications from past findings.

The finding of having an initial increase in elective care, such as outpatient appointments and ongoing management in terms of primary care, aligns with past findings of social prescribing being linked to improved confidence in management of long-term condition(s).^25^ This, paired with a longer term reduction in general practitioner use, aligns with the reasoning for the introduction of the scheme.

There are several key strengths that overcome weaknesses from previous studies. The first is the use of large national electronic healthcare records. We compare 168,434 patients referred to social prescribing with 1,310,723 matched patients who were not referred, which means our models have high statistical power and precision. Our models controlled for known indicators of inequalities in access to social prescribing link workers, these were regions of England and deprivation level.^3^^,^^4^ Next, we had a long pre-period to track appointment rates and an adequate follow-up period to determine the impact of referral. Our referral status was not self-reported, meaning there was no self-reporting bias. The impact and investment fund,^17^ which incentivised use of a specific SNOMED code to indicate referral to social prescribing, meant identification of referrals was likely to be the same across practices. The guidance stated that this code should only be used to indicate a referral, not an offer. This gave us some certainty that we were capturing an interaction with a social prescribing link worker.

We identified referral from the SNOMED code–*Referral to social prescribing*. This does not indicate take-up, fidelity, or a patient's readiness to engage.^26^ This is not recorded in CPRD; this means our results can only be interpreted as referrals to social prescribing, not engagement. There is no incentive for further coding of records, such as a *review of the social prescribing plan* or *social prescribing plan complete*, to indicate uptake and completion. For future research, there should be better integration between social prescribing platforms and electronic healthcare records, as currently this is not available, so the impact of referrals is the current best approach. Alternatively, evaluating a subgroup of general practices that actively use these additional SNOMED codes could help explore variation in the effects on outcomes. Furthermore, this *Referral to social prescribing service* code was incentivised through a point-based system, meaning higher referrals led to higher reimbursement (upper limit 1.2% of patients for max payment); this could lead to inappropriate referrals, particularly towards the end of the financial year. Previous studies have shown that financial incentives in primary care can change behaviour towards the end of the reporting period.^27^

We use a matching process that only takes into account measurable differences between referred and non-referred patients. As social prescribing aims to address complex social needs, there are likely to be additional unmeasured differences between these patient groups. This means, despite adjusting for the measured characteristics in our models, our matching approach does not fully account for all differences and there remains the possibility of unmeasured confounding. This means that we cannot be fully certain why these differences in healthcare use and costs occurred. Future work should explore how to account for these unmeasured differences and attribute the resulting effects of referrals to social prescribing on outcomes.

We were unable to assess the impact of referrals to social prescribing on emergency department attendances. The data was only available up to March 2020, which meant we could only estimate the impact for a small number of patients referred to social prescribing and only in the short term (6 months follow-up). However, given that social prescribing link workers are increasingly supporting patients with complex social needs,^28^ the main impacts may not be on emergency care utilisation.

Social prescribing was introduced to increase personalised care within primary care and widen support for patients with complex needs. Part of this universal personalised care model^6^ had expected benefits for the healthcare system, including reductions in GP contacts and emergency admissions. However, this does not necessarily mean there would be a reduction in overall contacts, as it might not be clinically appropriate and, therefore, for reduced demand and cost savings to occur. We also know from research into the inverse care law that people with complex needs who live in more deprived areas can face worse access, quality and experience of NHS services.^29^ In this context, link workers may be helping people with complex needs access and engage with existing health services.^30^ However, we do find a disproportionate number of patients from less deprived areas being referred to social prescribing compared to more deprived areas, which raises equity concerns. Past findings have highlighted that patients value having social prescribing link workers who they can trust and understand their wider needs, which aids with fidelity to the scheme.^31^ Interactions with a link worker to discuss their needs over the 6 to 12 appointments recommended by the NHS (though in practice this has been estimated to be around 4 appointments^32^), each lasting up to an hour, could be more appropriate than a 10-min appointment with clinical staff. The scheme has been found to improve satisfaction with primary care services,^25^ which could be a more appropriate aim than reducing healthcare use.

Referrals to social prescribing has impacted patients’ healthcare use with evidence of directing patients away from general practitioner appointments in the longer term. In the short term, we find evidence of an initial increase in use of healthcare that may be consistent with addressing unmet needs. This suggests that social prescribing may help patients manage non-clinical issues more effectively, leading to more appropriate and sustained use of healthcare services over time. This, paired with evidence of improved patient experience related to the rollout of link workers,^25^ highlights the scheme has been successful in delivering a *universal personalised care* model.^6^

## Contributors

AW, LM, JW, MS and PW conceptualised this study. AW, LM and PW gained data access. AW cleaned and analysed the data. AW and LM accessed and verified the underlying data. AW, LM, MS, JW, MB, SM and PW assessed the methods and interpreted the results. AW wrote the original manuscript. All authors contributed to writing (review and editing). PW, MS, LM, JW and SM gained funding for the study. All authors approved the final manuscript and accepted the responsibility for the decision to submit it for publication.

## Data sharing statement

The original data from this study is from Clinical Practice Research Datalink (CPRD) Aurum. Data cannot be shared publicly because they are not publicly available. CPRD may provide researchers with data following completion of their approvals and ethics process: https://www.cprd.com/research-applications.

## Declaration of interests

We declare no competing interests.

## Appendix A Supplementary data

Supplementary Files Updated
