# Supplementary material for: Changes in healthcare use and cost associated with referrals to social prescribing link workers in England: a matched case–control cohort study
Source: eClinicalMedicine. 2026 Aug 6;99:104102. doi: 10.1016/j.eclinm.2026.104102 (PMC13616562; doi:10.1016/j.eclinm.2026.104102)
Supplement: Supplementary file 1 [file mmc1.docx]

**Supplementary files**

**Supplementary Tables**

**Supplementary Table 1: Primary Care Costs from 2022 Unit Costs**

| **Role** | **2022 Unit Cost (£, 15 min)** | **Source / Notes** |
| --- | --- | --- |
| General Practitioner | ~£41 (per 10 min consult) | Table 9.4.2 |
| Practice Nurse | £13.00 (15 min, £52/hr incl. quals) | Table 9.3.1 |
| Healthcare Assistant | £12.00 (15 min, Band 3, ~£48/hr) | Table 12.3 (AfC Band 3) |
| Pharmacist | £28.00 (15 min, Band 8a, ~£112/hr) | Table 12.3 (AfC Band 8a) |
| Physician Associate | £27.00 (mapped to Band 7, ~£108/hr) | Table 12.3 (AfC Band 7) |
| Physiotherapist | £41.00 (Band 6/7 physio, ~£164/hr) | Table 12.3 (AfC Band 6–7) |
| Paramedic | £32.00 (Band 6, ~£128/hr) | Table 12.3 (AfC Band 6) |
| Phlebotomist | £6.00 (Band 2, ~£24/hr) | Table 12.3 (AfC Band 2) |
| Podiatrist | £33.00 (Band 6, ~£132/hr) | Table 12.3 (AfC Band 6) |
| Therapist | £36–45 (Band 6–7, £144–180/hr) | Table 12.3 (AfC Band 6–7) |
| Advanced Practitioner | £50.00 (Band 8b, ~£200/hr) | Table 12.3 (AfC Band 8b) |
| Counsellor | £67.00 (Band 7 psych/counsellor, ~£268/hr) | Table 12.3 (AfC Band 7) |
| Other | ~£40.00 (assumed avg of Bands 6–7) | Assumption |

**Source: PSSRU 2022 Unit Costs**

**Supplementary Table 2: Outpatient costs from 2022/23 Tariff**

| **Treatment function code** | **Treatment function description** | **WF01A  Follow Up Attendance -  Single Professional** | **WF01B  First Attendance -  Single Professional** |
| --- | --- | --- | --- |
| 100 | General Surgery Service | 78 | 181 |
| 101 | Urology Service | 71 | 145 |
| 103 | Breast Surgery Service | 79 | 187 |
| 104 | Colorectal Surgery Service | 68 | 157 |
| 105 | Hepatobiliary and Pancreatic Surgery Service | 115 | 269 |
| 106 | Upper Gastrointestinal Surgery Service | 80 | 187 |
| 107 | Vascular Surgery Service | 85 | 194 |
| 108 | Spinal Surgery Service | 88 | 222 |
| 110 | Trauma and Orthopaedic Service | 67 | 169 |
| 120 | Ear Nose and Throat Service | 57 | 133 |
| 130 | Ophthalmology Service | 64 | 142 |
| 140 | Oral Surgery Service | 69 | 142 |
| 143 | Orthodontic Service | 76 | 192 |
| 144 | Maxillofacial Surgery Service | 71 | 151 |
| 160 | Plastic Surgery Service | 60 | 152 |
| 170 | Cardiothoracic Surgery Service | 123 | 286 |
| 171 | Paediatric Surgery Service | 115 | 191 |
| 172 | Cardiac Surgery Service | 123 | 286 |
| 173 | Thoracic Surgery Service | 120 | 303 |
| 190 | Anaesthetic Service | 70 | 173 |
| 191 | Pain Management Service | 87 | 216 |
| 211 | Paediatric Urology Service | 86 | 145 |
| 214 | Paediatric Trauma and Orthopaedic Service | 94 | 169 |
| 215 | Paediatric Ear Nose and Throat Service | 68 | 127 |
| 216 | Paediatric Ophthalmology Service | 82 | 139 |
| 217 | Paediatric Oral and Maxillofacial Surgery Service | 135 | 191 |
| 219 | Paediatric Plastic Surgery Service | 96 | 145 |
| 223 | Paediatric Epilepsy Service | 207 | 266 |
| 251 | Paediatric Gastroenterology Service | 162 | 279 |
| 252 | Paediatric Endocrinology Service | 172 | 332 |
| 253 | Paediatric Clinical Haematology Service | 189 | 400 |
| 257 | Paediatric Dermatology Service | 104 | 156 |
| 258 | Paediatric Respiratory Medicine Service | 148 | 278 |
| 263 | Paediatric Diabetes Service | 226 | 522 |
| 300 | General Internal Medicine Service | 109 | 197 |
| 301 | Gastroenterology Service | 85 | 215 |
| 302 | Endocrinology Service | 99 | 246 |
| 303 | Clinical Haematology Service | 131 | 288 |
| 306 | Hepatology Service | 153 | 291 |
| 307 | Diabetes Service | 106 | 134 |
| 320 | Cardiology Service | 94 | 179 |
| 321 | Paediatric Cardiology Service | 131 | 213 |
| 329 | Transient Ischaemic Attack Service | - | 402 |
| 330 | Dermatology Service | 74 | 134 |
| 340 | Respiratory Medicine Service | 100 | 225 |
| 341 | Respiratory Physiology Service | 80 | 171 |
| 350 | Infectious Diseases Service | 154 | 375 |
| 361 | Renal Medicine Service | 133 | 183 |
| 370 | Medical Oncology Service | 135 | 298 |
| 410 | Rheumatology Service | 96 | 283 |
| 420 | Paediatric Service | 156 | 232 |
| 430 | Elderly Medicine Service | 145 | 308 |
| 502 | Gynaecology Service | 78 | 167 |
| 503 | Gynaecological Oncology Service | 77 | 159 |
| 800 | Clinical Oncology Service | 132 | 284 |
| 812 | Diagnostic Imaging Service | 0 | 0 |

**Source: NHS 2022-2023 Tariff**

**Supplementary Table 3: Rationale for inclusion in exact matching (based on NHS Social Prescribing Scheme)**

| **Covariate(s)** | **Rationale** |
| --- | --- |
| Morbidity Measure | Social prescribing is aimed at patients with one or more long term conditions. Inclusion of the number of long-term conditions is a predictor of being referred. |
| Diagnosed with Anxiety or Depression | Social prescribing is aimed at patients who require support for their mental health. |
| Number of primary care appointments in the six months prior to referral | Previous primary care use, particularly if that has been an increase is likely to lead to an increase in probability of being referred. We want to compare like-for-like. |
| Month of referral | We want to match on patients who have the same index month, so comparing the same time periods. |
| Year of referral | We want to match on patients who have the same index year, so comparing the same time periods. |

**Supplementary Table 4: Rationale for Covariate Inclusion**

| **Covariate(s)** | **Rationale** |
| --- | --- |
| Lag of outcome of two years prior | Previous healthcare use/cost is likely to predict future use, controlling for this reduces bias from a known predictor of future need |
| Age – linear and quadratic term | Older patients use more healthcare; however, this relationship may not be linear, so we include a quadratic term to adjust for this. |
| Sex (Female =1) | Mortality and morbidity vary by sex; this relates to healthcare. Similarly, health seeking behaviour varies by sex. |
| Ethnicity | Prevalence of health conditions vary by ethnicity but also interaction with healthcare. This can affect care received. |
| Deprivation – Income domain | Patients in more deprived areas are linked to worse access to healthcare, experience and outcomes. |
| Morbidity Measure | Number of long-term conditions is an indicator of potential use of healthcare services, as they require ongoing treatment and medication from healthcare providers |
| Region | In addition to deprivation, there are regional inequalities in staffing, access to healthcare and outcomes. This variable in parts aim to adjust for broad NHS Regions, however they do not map perfectly. |
| Month of referral | There is seasonality throughout the year, we aim to adjust for that with month fixed effects. Particularly for the 6 month follow up, we want to adjust patients whose follow up crosses the winter period. |
| Year of referral | There are ongoing changes in healthcare use annually and also changes in the number of patients referred to social prescribing. Year fixed effects aim to adjust for both these factors. |

**Supplementary Table 5: Unadjusted descriptive statistics of patient characteristics for unreferred patients (N=1,359,480)**

| **Covariate** | **Mean** | **(SD)/Freq** |
| --- | --- | --- |
| **Age** | 57·03 | (19·65) |
| **Female** | 62·8% | 853,107 |
| **Anxiety or Depression** | 28·3% | 384,539 |
| **Morbidity Measure** |  |  |
| No LTC | 24·4% | 331,443 |
| 1 LTC | 24·4% | 331,210 |
| 2 LTC | 18.0% | 244,183 |
| 3 LTC | 12·3% | 166,744 |
| 4 or more LTCs | 21.0% | 285,900 |
| **Ethnicity** |  |  |
| Asian | 7·6% | 103,092 |
| Black | 4.0% | 53,785 |
| Mixed | 1·2% | 15,970 |
| White | 85.0% | 1,155,437 |
| Other | 0·3% | 4,343 |
| Unknown | 2.0% | 26,853 |
| **IMD Decile - Income** |  |  |
| 1 = Most | 10·8% | 146,507 |
| 2 | 10·8% | 146,964 |
| 3 | 10·7% | 145,045 |
| 4 | 10.0% | 136,527 |
| 5 | 9·9% | 134,167 |
| 6 | 9·7% | 131,310 |
| 7 | 10·1% | 137,272 |
| 8 | 9·6% | 131,100 |
| 9 | 9·6% | 131,150 |
| 10 = Least | 8·8% | 119,438 |
| **Region** |  |  |
| East Midlands | 2·2% | 30,220 |
| East of England | 4·4% | 60,302 |
| London | 17·6% | 238,648 |
| North East | 3·6% | 48,818 |
| North West | 18·2% | 247,433 |
| South East | 20·5% | 278,022 |
| South West | 12·8% | 174,126 |
| West Midlands | 17·2% | 233,688 |
| Yorkshire and The Humber | 3·5% | 48,223 |

**Supplementary Table 6: Descriptive statistics of patient characteristics for unmatched patients (N=67,255)**

|  | **Referred** | | **Non-referred** | |
| --- | --- | --- | --- | --- |
|  | %/Mean | (SD)/Freq | %/Mean | (SD)/Freq |
| Patients | 5,671 | | 61,584 | |
| **Age** | 59·34 | (20·04) | 54·65 | (19·93) |
| **Female** | 66·3% | 3,762 | 63·7% | 39,228 |
| **Male** | 33·7% | 1,909 | 36·3% | 22,356 |
| **Anxiety or Depression** | 66·2% | 3,755 | 32·2% | 19,848 |
| **Morbidity Measure** |  |  |  |  |
| 0 LTC | 5·5% | 314 | 35·4% | 21,798 |
| 1 LTC | 14·6% | 827 | 21·4% | 13,192 |
| 2 LTCs | 18·3% | 1,039 | 16·1% | 9,893 |
| 3 LTCs | 19·7% | 1,119 | 14·2% | 8,725 |
| 4 or more LTCs | 41·8% | 2,372 | 13.0% | 7,976 |
| **Ethnicity** |  |  |  |  |
| Asian | 7·4% | 418 | 7·6% | 4,679 |
| Black | 5·6% | 318 | 3·9% | 2,402 |
| Mixed | 1·6% | 90 | 1·2% | 764 |
| White | 83·9% | 4,757 | 84·8% | 52,206 |
| Other | 0·3% | 18 | 0·3% | 178 |
| Unknown | 1·2% | 70 | 2·2% | 1,355 |
| **Income deprivation decile** |  |  |  |  |
| 1 = Most | 8.0% | 456 | 10·9% | 6,703 |
| 2 | 8·6% | 488 | 11.0% | 6,764 |
| 3 | 8·8% | 500 | 10·7% | 6,610 |
| 4 | 7·8% | 445 | 10·5% | 6,488 |
| 5 | 8·7% | 494 | 9·7% | 5,990 |
| 6 | 10·4% | 592 | 9·6% | 5,926 |
| 7 | 11·3% | 643 | 10·1% | 6,210 |
| 8 | 12·4% | 704 | 9·7% | 5,952 |
| 9 | 12·2% | 691 | 9·4% | 5,785 |
| 10 = Least | 11·6% | 658 | 8·4% | 5,156 |
| **Region** |  |  |  |  |
| North East | 1.0% | 59 | 2·5% | 1,513 |
| North West | 4·1% | 230 | 4·5% | 2,757 |
| Yorkshire and Humberside | 21·5% | 1,220 | 16·8% | 10,336 |
| East Midlands | 3·8% | 216 | 3·3% | 2,010 |
| West Midlands | 13·8% | 784 | 16·7% | 10,311 |
| East of England | 21·6% | 1,226 | 21·3% | 13,147 |
| London | 15·7% | 888 | 14.0% | 8,648 |
| South East | 15·1% | 854 | 17·3% | 10,654 |
| South West | 3·4% | 194 | 3·6% | 2,208 |

Note: Post-hoc analysis

**Supplementary Table 7: Cost in £ of healthcare use pre- and post- referral by referral status**

|  |  | **Referred** | | | | **Non-referred** | | | |
| --- | --- | --- | --- | --- | --- | --- | --- | --- | --- |
|  |  | Pre-referral | | Post-referral | | Pre-referral* | | Post-referral* | |
|  | ***Time Frame*** | **Mean** | **(SD)** | **Mean** | **(SD)** | **Mean** | **(SD)** | **Mean** | **(SD)** |
| **Secondary and Primary Care** | *6 months* | 1062 | (3168) | 1022 | (3066) | 799 | (2669) | 891 | (2854) |
| **Primary Care** | *6 months* | 250 | (249) | 251 | (223) | 185 | (209) | 239 | (212) |
| *General Practitioner* | *6 months* | 192 | (215) | 201 | (200) | 142 | (181) | 186 | (191) |
| **Secondary Care** | *6 months* | 1055 | (3167) | 1014 | (3065) | 792 | (2668) | 885 | (2853) |
| *Outpatients* | *6 months* | 219 | (732) | 213 | (662) | 178 | (583) | 185 | (582) |
| *Admitted Patient Care* | *6 months* | 836 | (2938) | 802 | (2847) | 613 | (2473) | 700 | (2658) |
| *Elective* | *6 months* | 217 | (1223) | 235 | (1279) | 194 | (1081) | 216 | (1227) |
| *Emergency* | *6 months* | 619 | (2578) | 566 | (2428) | 420 | (2134) | 485 | (2259) |
| **Secondary and Primary Care** | *1 year* | 1951 | (4772) | 1836 | (4687) | 1560 | (4185) | 1630 | (4343) |
| **Primary Care** | *1 year* | 457 | (417) | 442 | (379) | 359 | (358) | 417 | (360) |
| *General Practitioner* | *1 year* | 350 | (358) | 355 | (337) | 272 | (306) | 324 | (318) |
| **Secondary Care** | *1 year* | 1937 | (4769) | 1822 | (4685) | 1549 | (4182) | 1616 | (4340) |
| Outpatients | *1 year* | 408 | (1180) | 401 | (1086) | 344 | (1002) | 350 | (978) |
| Admitted Patient Care | *1 year* | 1529 | (4348) | 1421 | (4269) | 1205 | (3794) | 1266 | (3961) |
| *Elective* | *1 year* | 414 | (1843) | 451 | (2076) | 382 | (1643) | 408 | (1829) |
| *Emergency* | *1 year* | 1115 | (3766) | 970 | (3476) | 823 | (3231) | 858 | (3312) |
| Observations |  | 168,699 | | | | 1,302,443 | | | |

Note: APC = admitted patient care. * referral date here is the index date, this is randomised to matched density of referrals across study period

**Supplementary Table 8: Unweighted healthcare use and cost pre- and post-referral for unreferred patients (N=1,359,480)**

|  | ***Time Frame*** | **Pre** | | **Post** | |
| --- | --- | --- | --- | --- | --- |
| **Use** | | | | | |
| **Secondary and Primary Care** | *6 months* | 4·45 | (6·44) | 4·56 | (6·56) |
| **Primary Care** | *6 months* | 3·32 | (4·58) | 3·41 | (4·69) |
| *General Practitioner* | *6 months* | 1·87 | (2·97) | 1·90 | (3·03) |
| **Secondary Care** | *6 months* | 1·13 | (3·49) | 1·15 | (3·53) |
| *Outpatients* | *6 months* | 0·91 | (2·80) | 0·93 | (2·82) |
| *Admitted Patient Care* | *6 months* | 0·21 | (1·49) | 0·22 | (1·52) |
| *Elective* | *6 months* | 0·13 | (1·39) | 0·13 | (1·42) |
| *Emergency* | *6 months* | 0·07 | (0·37) | 0·07 | (0·37) |
| **Secondary and Primary Care** | *1 year* | 9·24 | (11·70) | 8·69 | (11·25) |
| **Primary Care** | *1 year* | 6·92 | (8·18) | 6·46 | (7·83) |
| *General Practitioner* | *1 year* | 3·81 | (5·16) | 3·66 | (5·00) |
| **Secondary Care** | *1 year* | 2·33 | (6·29) | 2·23 | (6·14) |
| Outpatients | *1 year* | 1·89 | (4·93) | 1·81 | (4·86) |
| Admitted Patient Care | *1 year* | 0·44 | (2·81) | 0·42 | (2·73) |
| *Elective* | *1 year* | 0·27 | (2·66) | 0·25 | (2·59) |
| *Emergency* | *1 year* | 0·15 | (0·58) | 0·14 | (0·58) |
| **Cost (£)** | | | | | |
| **Secondary and Primary Care** | *6 months* | 427 | (1903) | 417 | (1906) |
| **Primary Care** | *6 months* | 106 | (151) | 105 | (149) |
| *General Practitioner* | *6 months* | 81 | (129) | 81 | (129) |
| **Secondary Care** | *6 months* | 423 | (1903) | 413 | (1905) |
| *Outpatients* | *6 months* | 100 | (411) | 99 | (411) |
| *Admitted Patient Care* | *6 months* | 324 | (1758) | 314 | (1761) |
| *Elective* | *6 months* | 115 | (828) | 115 | (904) |
| *Emergency* | *6 months* | 209 | (1480) | 199 | (1437) |
| **Secondary and Primary Care** | *1 year* | 862 | (3030) | 820 | (2986) |
| **Primary Care** | *1 year* | 213 | (259) | 205 | (254) |
| *General Practitioner* | *1 year* | 161 | (218) | 159 | (218) |
| **Secondary Care** | *1 year* | 855 | (3027) | 814 | (2984) |
| Outpatients | *1 year* | 200 | (724) | 196 | (705) |
| Admitted Patient Care | *1 year* | 655 | (2730) | 617 | (2698) |
| *Elective* | *1 year* | 235 | (1275) | 229 | (1346) |
| *Emergency* | *1 year* | 420 | (2261) | 388 | (2187) |

**Supplementary Table 9: Healthcare use pre- and post-referral for unmatched patients (N=67,255)**

|  |  | **Referred** | | | | **Non-referred** | | | |
| --- | --- | --- | --- | --- | --- | --- | --- | --- | --- |
|  |  | Pre-referral | | Post-referral | | Pre-referral* | | Post-referral* | |
|  | ***Time Frame*** | **Mean** | **(SD)** | **Mean** | **(SD)** | **Mean** | **(SD)** | **Mean** | **(SD)** |
| **Secondary and Primary Care** | *6 months* | 31·07 | (19·08) | 23·22 | (19·95) | 7·00 | (11·37) | 5·49 | (8·91) |
| **Primary Care** | *6 months* | 26·72 | (15·56) | 19·23 | (15·92) | 5·64 | (9·39) | 4·24 | (7·05) |
| *General Practitioner* | *6 months* | 15·79 | (11·50) | 11·25 | (11·05) | 3·03 | (5·36) | 2·30 | (4·01) |
| **Secondary Care** | *6 months* | 4·35 | (8·64) | 3·99 | (10·08) | 1·36 | (3·91) | 1·25 | (3·71) |
| *Outpatients* | *6 months* | 3·43 | (7·97) | 3·23 | (9·59) | 1·10 | (3·30) | 1·01 | (3·06) |
| *Admitted Patient Care* | *6 months* | 0·92 | (2·09) | 0·75 | (2·08) | 0·26 | (1·25) | 0·23 | (1·43) |
| *Elective* | *6 months* | 0·34 | (1·58) | 0·30 | (1·74) | 0·14 | (1·07) | 0·13 | (1·30) |
| *Emergency* | *6 months* | 0·55 | (1·13) | 0·43 | (1·03) | 0·11 | (0·48) | 0·08 | (0·42) |
| **Secondary and Primary Care** | *1 year* | 41·32 | (33·68) | 49·42 | (32·18) | 10·68 | (15·70) | 12·09 | (18·18) |
| **Primary Care** | *1 year* | 34·09 | (27·85) | 41·79 | (26·37) | 8·23 | (12·27) | 9·54 | (14·81) |
| *General Practitioner* | *1 year* | 20·05 | (19·32) | 24·84 | (19·05) | 4·48 | (6·87) | 5·16 | (8·27) |
| **Secondary Care** | *1 year* | 7·23 | (14·32) | 7·63 | (14·16) | 2·45 | (6·51) | 2·55 | (6·42) |
| Outpatients | *1 year* | 5·85 | (13·29) | 6·05 | (12·92) | 1·99 | (5·20) | 2·07 | (5·39) |
| Admitted Patient Care | *1 year* | 1·38 | (3·53) | 1·58 | (3·82) | 0·46 | (2·75) | 0·48 | (2·12) |
| *Elective* | *1 year* | 0·56 | (2·90) | 0·62 | (3·14) | 0·27 | (2·55) | 0·26 | (1·88) |
| *Emergency* | *1 year* | 0·78 | (1·61) | 0·90 | (1·69) | 0·16 | (0·68) | 0·19 | (0·70) |
| Observations |  | 5671 | | | | 61584 | | | |

Note: APC – admitted patient care * referral date here is the index date, this is randomised to matched density of referrals across study period. Post-hoc analysis

**Supplementary Table 10: Healthcare cost pre- and post-referral for unmatched patients (N=67,255)**

|  |  | **Referred** | | | | **Non-referred** | | | |
| --- | --- | --- | --- | --- | --- | --- | --- | --- | --- |
|  |  | Pre-referral | | Post-referral | | Pre-referral* | | Post-referral* | |
|  | ***Time Frame*** | **Mean** | **(SD)** | **Mean** | **(SD)** | **Mean** | **(SD)** | **Mean** | **(SD)** |
| **Secondary and Primary Care** | *6 months* | 1698 | (3917) | 2052 | (4413) | 476 | (2076) | 538 | (2200) |
| **Primary Care** | *6 months* | 612 | (521) | 862 | (520) | 132 | (209) | 178 | (285) |
| *General Practitioner* | *6 months* | 480 | (472) | 685 | (494) | 100 | (173) | 135 | (235) |
| **Secondary Care** | *6 months* | 1671 | (3915) | 2033 | (4412) | 470 | (2074) | 534 | (2199) |
| *Outpatients* | *6 months* | 322 | (785) | 348 | (762) | 110 | (434) | 117 | (442) |
| *Admitted Patient Care* | *6 months* | 1349 | (3688) | 1685 | (4149) | 360 | (1921) | 417 | (2033) |
| *Elective* | *6 months* | 275 | (1149) | 388 | (1569) | 123 | (885) | 145 | (976) |
| *Emergency* | *6 months* | 1074 | (3458) | 1297 | (3686) | 236 | (1625) | 271 | (1668) |
| **Secondary and Primary Care** | *1 year* | 3101 | (6040) | 3405 | (6250) | 926 | (3327) | 992 | (3388) |
| **Primary Care** | *1 year* | 1083 | (912) | 1352 | (876) | 254 | (359) | 302 | (443) |
| *General Practitioner* | *1 year* | 848 | (820) | 1080 | (820) | 193 | (293) | 231 | (364) |
| **Secondary Care** | *1 year* | 3067 | (6035) | 3364 | (6245) | 918 | (3324) | 982 | (3383) |
| Outpatients | *1 year* | 575 | (1273) | 619 | (1261) | 215 | (756) | 219 | (722) |
| Admitted Patient Care | *1 year* | 2492 | (5617) | 2745 | (5769) | 703 | (3021) | 764 | (3074) |
| *Elective* | *1 year* | 534 | (1809) | 661 | (2136) | 248 | (1432) | 268 | (1425) |
| *Emergency* | *1 year* | 1958 | (5161) | 2084 | (5065) | 455 | (2448) | 495 | (2516) |
| Observations |  | 5671 | | | | 61584 | | | |

Note: APC – admitted patient care * referral date here is the index date, this is randomised to matched density of referrals across study period. Post-hoc analysis

**Supplementary Table 11: Healthcare use pre- and post-referral by referral status for the sub-sample where observations were matched on the 1-year prior primary care use, in addition to matching variables used for the sample in Tables 2 and 3.**

|  |  | **Referred** | | | | **Non-referred** | | | |
| --- | --- | --- | --- | --- | --- | --- | --- | --- | --- |
|  |  | Pre-referral | | Post-referral | | Pre-referral* | | Post-referral* | |
|  | ***Time Frame*** | **Mean** | **(SD)** | **Mean** | **(SD)** | **Mean** | **(SD)** | **Mean** | **(SD)** |
| **Secondary and Primary Care** | *6 months* | 7·55 | (7·23) | 7·73 | (7·82) | 7·30 | (6·67) | 6·98 | (7·17) |
| **Primary Care** | *6 months* | 5·53 | (4·28) | 5·67 | (5·00) | 5·55 | (4·30) | 5·20 | (4·81) |
| *General Practitioner* | *6 months* | 3·32 | (3·19) | 3·20 | (3·48) | 3·13 | (3·13) | 2·92 | (3·36) |
| **Secondary Care** | *6 months* | 2·02 | (5·19) | 2·05 | (5·30) | 1·75 | (4·28) | 1·77 | (4·39) |
| *Outpatients* | *6 months* | 1·57 | (4·06) | 1·61 | (4·20) | 1·40 | (3·33) | 1·43 | (3·41) |
| *Admitted Patient Care* | *6 months* | 0·45 | (2·48) | 0·44 | (2·46) | 0·34 | (2·04) | 0·35 | (2·10) |
| *Elective* | *6 months* | 0·28 | (2·37) | 0·27 | (2·34) | 0·21 | (1·94) | 0·22 | (2·00) |
| *Emergency* | *6 months* | 0·16 | (0·55) | 0·16 | (0·57) | 0·12 | (0·45) | 0·12 | (0·47) |
| **Secondary and Primary Care** | *1 year* | 14·31 | (12·89) | 14·06 | (12·98) | 13·97 | (12·32) | 13·59 | (12·10) |
| **Primary Care** | *1 year* | 10·42 | (7·84) | 10·16 | (7·71) | 10·44 | (7·84) | 10·22 | (7·77) |
| *General Practitioner* | *1 year* | 5·88 | (5·43) | 6·09 | (5·52) | 5·75 | (5·45) | 5·78 | (5·43) |
| **Secondary Care** | *1 year* | 3·89 | (8·90) | 3·90 | (9·05) | 3·53 | (7·83) | 3·37 | (7·57) |
| Outpatients | *1 year* | 3·07 | (6·85) | 3·03 | (6·85) | 2·84 | (5·98) | 2·72 | (5·80) |
| Admitted Patient Care | *1 year* | 0·83 | (4·47) | 0·87 | (4·63) | 0·69 | (3·85) | 0·65 | (3·77) |
| *Elective* | *1 year* | 0·51 | (4·29) | 0·55 | (4·45) | 0·43 | (3·68) | 0·40 | (3·61) |
| *Emergency* | *1 year* | 0·30 | (0·85) | 0·29 | (0·83) | 0·24 | (0·73) | 0·22 | (0·69) |
| Observations |  | 120,159 | | | | 808,238 | | | |

Note: APC – admitted patient care * referral date here is the index date, this is randomised to matched density of referrals across study period

**Supplementary Table 12: Healthcare cost (£) pre- and post-referral by referral status for the sub-sample where observations were matched on the 1-year prior primary care use, in addition to matching variables used for the sample in Tables 2 and Supplementary Table 5.**

|  |  | **Referred** | | | | **Non-referred** | | | |
| --- | --- | --- | --- | --- | --- | --- | --- | --- | --- |
|  |  | Pre-referral | | Post-referral | | Pre-referral* | | Post-referral* | |
|  | ***Time Frame*** | **Mean** | **(SD)** | **Mean** | **(SD)** | **Mean** | **(SD)** | **Mean** | **(SD)** |
| **Secondary and Primary Care** | *6 months* | 882 | (2870) | 846 | (2751) | 699 | (2458) | 661 | (2352) |
| **Primary Care** | *6 months* | 179 | (168) | 181 | (151) | 162 | (161) | 175 | (148) |
| *General Practitioner* | *6 months* | 137 | (148) | 143 | (138) | 124 | (143) | 136 | (135) |
| **Secondary Care** | *6 months* | 877 | (2869) | 840 | (2751) | 694 | (2458) | 655 | (2351) |
| *Outpatients* | *6 months* | 191 | (691) | 187 | (628) | 160 | (532) | 158 | (524) |
| *Admitted Patient Care* | *6 months* | 686 | (2653) | 653 | (2544) | 533 | (2281) | 498 | (2175) |
| *Elective* | *6 months* | 190 | (1124) | 208 | (1195) | 178 | (1042) | 180 | (1136) |
| *Emergency* | *6 months* | 496 | (2318) | 446 | (2150) | 355 | (1954) | 317 | (1764) |
| **Secondary and Primary Care** | *1 year* | 1626 | (4274) | 1562 | (4310) | 1394 | (3853) | 1259 | (3651) |
| **Primary Care** | *1 year* | 327 | (262) | 333 | (267) | 321 | (260) | 324 | (263) |
| *General Practitioner* | *1 year* | 249 | (230) | 265 | (240) | 243 | (231) | 251 | (236) |
| **Secondary Care** | *1 year* | 1615 | (4272) | 1552 | (4308) | 1384 | (3851) | 1248 | (3649) |
| Outpatients | *1 year* | 355 | (1110) | 360 | (1049) | 315 | (928) | 306 | (895) |
| Admitted Patient Care | *1 year* | 1260 | (3881) | 1192 | (3906) | 1068 | (3503) | 942 | (3307) |
| *Elective* | *1 year* | 365 | (1705) | 410 | (2035) | 355 | (1564) | 352 | (1781) |
| *Emergency* | *1 year* | 895 | (3327) | 782 | (3085) | 714 | (2985) | 591 | (2615) |
| Observations |  | 120,159 | | | | 808,238 | | | |

Note: APC = * referral date here is the index date, this is randomised to matched density of referrals across study period

**Supplementary Table 13: Unweighted and unadjusted Models of Table 4: Regression results for main coefficient of interest *referrals to social prescribing* on outcomes – healthcare use and cost for 6 months and 1 year follow-up**

|  | **(1)** | **(2)** | **(3)** | **(4)** | **(5)** | **(6)** | **(7)** | **(8)** |
| --- | --- | --- | --- | --- | --- | --- | --- | --- |
|  | Primary Care and Hospital | All primary care | General Practitioner | All Hospital | Outpatient | Admitted Patient Care | Admitted Patient Care - Emergency | Admitted Patient Care - Elective |
| **Appointment** |  |  |  |  |  |  |  |  |
| *6 months post* | 6·175*** | 4·845*** | 2·807*** | 1·331*** | 1·025*** | 0·306*** | 0·163*** | 0·142*** |
|  | (5·907 to 6·444) | (4·610 to 5·079) | (2·676 to 2·937) | (1·251 to 1·411) | (0·960 to 1·091) | (0·276 to 0·335) | (0·136 to 0·190) | (0·134 to 0·150) |
|  |  |  |  |  |  |  |  |  |
| *1 year post* | 10·150*** | 7·825*** | 5·026*** | 2·326*** | 1·754*** | 0·572*** | 0·323*** | 0·240*** |
|  | (9·742 to 10·559) | (7·497 to 8·152) | (4·810 to 5·243) | (2·173 to 2·479) | (1·643 to 1·866) | (0·509 to 0·634) | (0·267 to 0·379) | (0·225 to 0·254) |
|  |  |  |  |  |  |  |  |  |
| **Cost** |  |  |  |  |  |  |  |  |
| *6 months post* | 640·218*** | 165·701*** | 135·068*** | 635·373*** | 117·659*** | 517·714*** | 125·357*** | 392·344*** |
|  | (597·844 to 682·591) | (158·765 to 172·636) | (129·091 to 141·046) | (593·067 to 677·679) | (109·453 to 125·866) | (480·025 to 555·402) | (114·717 to 135·997) | (360·685 to 424·002) |
|  |  |  |  |  |  |  |  |  |
| *1 year post* | 1068·562*** | 267·147*** | 218·775*** | 1060·738*** | 211·629*** | 849·108*** | 229·170*** | 619·892*** |
|  | (997·090 to 1140·034) | (256·188 to 278·106) | (209·309 to 228·241) | (989·393 to 1132·083) | (196·116 to 227·142) | (788·158 to 910·059) | (207·146 to 251·194) | (573·557 to 666·226) |

**Note:** N= 1,533,796, 95% Confidence Intervals based on clustered standard errors at general practice (N=1,432) level in parentheses. * p<0·05, ** p<0·01, *** p<0·001.

**Supplementary Table 14: Unadjusted Models of Table 4: Regression results for main coefficient of interest *referrals to social prescribing* on outcomes – healthcare use and cost for 6 months and 1 year follow-up**

|  | **(1)** | **(2)** | **(3)** | **(4)** | **(5)** | **(6)** | **(7)** | **(8)** |
| --- | --- | --- | --- | --- | --- | --- | --- | --- |
|  | Primary Care and Hospital | All primary care | General Practitioner | All Hospital | Outpatient | Admitted Patient Care | Admitted Patient Care - Emergency | Admitted Patient Care - Elective |
| **Appointment** |  |  |  |  |  |  |  |  |
| *6 months post* | 2·423*** | 1·978*** | 1·182*** | 0·445*** | 0·324*** | 0·121*** | 0·056*** | 0·067*** |
|  | (2·176 to 2·671) | (1·764 to 2·193) | (1·065 to 1·299) | (0·369 to 0·522) | (0·265 to 0·383) | (0·089 to 0·153) | (0·027 to 0·085) | (0·059 to 0·075) |
|  |  |  |  |  |  |  |  |  |
| *1 year post* | 0·801*** | 0·278 | 0·702*** | 0·524*** | 0·349*** | 0·175*** | 0·114*** | 0·059*** |
|  | (0·428 to 1·174) | (-0·007 to 0·562) | (0·516 to 0·889) | (0·371 to 0·676) | (0·244 to 0·455) | (0·108 to 0·241) | (0·054 to 0·173) | (0·043 to 0·074) |
|  |  |  |  |  |  |  |  |  |
| **Cost** |  |  |  |  |  |  |  |  |
| *6 months post* | 131·459*** | 11·768*** | 14·997*** | 129·480*** | 28·106*** | 101·374*** | 19·578*** | 81·791*** |
|  | (86·866 to 176·051) | (5·924 to 17·613) | (9·917 to 20·078) | (84·951 to 174·010) | (19·697 to 36·516) | (61·634 to 141·114) | (8·125 to 31·031) | (48·210 to 115·372) |
|  |  |  |  |  |  |  |  |  |
| *1 year post* | 206·156*** | 25·815*** | 30·448*** | 205·878*** | 50·894*** | 154·984*** | 42·607*** | 112·342*** |
|  | (130·497 to 281·815) | (16·415 to 35·216) | (22·301 to 38·595) | (130·343 to 281·413) | (34·994 to 66·793) | (90·231 to 219·738) | (19·510 to 65·703) | (62·543 to 162·141) |

**Note:** N= 1,471,142, 95% Confidence Intervals based on clustered standard errors at general practice (N=1,432) level in parentheses. * p<0·05, ** p<0·01, *** p<0·001. Models include weights from exact matching

**Supplementary Table 15: Unweighted Models of Table 4: Regression results for main coefficient of interest *referrals to social prescribing* on outcomes – healthcare use and cost for 6 months and 1 year follow-up**

|  | **(1)** | **(2)** | **(3)** | **(4)** | **(5)** | **(6)** | **(7)** | **(8)** |
| --- | --- | --- | --- | --- | --- | --- | --- | --- |
|  | Primary Care and Hospital | All primary care | General Practitioner | All Hospital | Outpatient | Admitted Patient Care | Admitted Patient Care - Emergency | Admitted Patient Care - Elective |
| **Appointment** |  |  |  |  |  |  |  |  |
| *6 months post* | 3·378*** | 3·011*** | 1·260*** | 0·491*** | 0·410*** | 0·089*** | 0·034*** | 0·078*** |
|  | (3·184 to 3·572) | (2·831 to 3·191) | (1·189 to 1·331) | (0·441 to 0·541) | (0·364 to 0·456) | (0·077 to 0·100) | (0·024 to 0·043) | (0·073 to 0·083) |
|  |  |  |  |  |  |  |  |  |
| *1 year post* | 3·295*** | 3·361*** | 0·733*** | 0·198*** | 0·139*** | 0·062*** | 0·024*** | 0·035*** |
|  | (3·066 to 3·523) | (3·155 to 3·567) | (0·689 to 0·776) | (0·165 to 0·231) | (0·110 to 0·169) | (0·052 to 0·071) | (0·017 to 0·032) | (0·030 to 0·039) |
|  |  |  |  |  |  |  |  |  |
| **Cost** |  |  |  |  |  |  |  |  |
| *6 months post* | 186·602*** | 65·363*** | 42·082*** | 185·240*** | 20·681*** | 164·129*** | 31·276*** | 133·377*** |
|  | (163·517 to 209·687) | (61·806 to 68·921) | (39·752 to 44·412) | (162·182 to 208·298) | (17·453 to 23·909) | (142·647 to 185·611) | (20·286 to 42·266) | (117·005 to 149·750) |
|  |  |  |  |  |  |  |  |  |
| *1 year post* | 166·129*** | 66·330*** | 32·531*** | 165·548*** | 16·452*** | 148·838*** | 39·244*** | 112·247*** |
|  | (143·766 to 188·491) | (62·049 to 70·611) | (30·589 to 34·474) | (143·223 to 187·873) | (12·927 to 19·977) | (128·163 to 169·512) | (25·480 to 53·008) | (98·485 to 126·009) |

**Note:** N= 1,533,796, 95% Confidence Intervals based on clustered standard errors at general practice (N=1,432) level in parentheses. * p<0·05, ** p<0·01, *** p<0·001. Models include covariates: outcome 2 years prior, age, age^2, female, ethnicity group, IMD – Income Domain Decile, government office region, and month and year fixed effects of referral date

**Supplementary Table 16: Coefficients from Models in Table 4 – healthcare use – 6 months follow-up**

|  | (1) | (2) | (3) | (4) | (5) | (6) | (7) | (8) |
| --- | --- | --- | --- | --- | --- | --- | --- | --- |
|  | Primary Care and Hospital | All primary care | General Practitioner | All Hospital | Outpatient | Admitted Patient Care | Admitted Patient Care - Elective | Admitted Patient Care - Emergency |
| 2 year Lag of Outcome | 2·252*** | 1·961*** | 0·914*** | 0·248*** | 0·196*** | 0·054*** | 0·009 | 0·055*** |
|  | (2·089 to 2·414) | (1·812 to 2·111) | (0·855 to 0·972) | (0·199 to 0·296) | (0·152 to 0·240) | (0·041 to 0·066) | (-0·003 to 0·020) | (0·051 to 0·059) |
| Age | 0·120*** | 0·090*** | 0·190*** | 0·193*** | 0·183*** | 0·211*** | 0·213*** | 0·132*** |
|  | (0·115 to 0·126) | (0·086 to 0·095) | (0·187 to 0·192) | (0·186 to 0·201) | (0·172 to 0·195) | (0·199 to 0·222) | (0·201 to 0·225) | (0·127 to 0·136) |
| Age^2 | 0·022*** | -0·027*** | -0·031*** | 0·038*** | 0·034*** | 0·005*** | 0·007*** | -0·004*** |
|  | (0·009 to 0·036) | (-0·038 to -0·016) | (-0·036 to -0·025) | (0·033 to 0·043) | (0·029 to 0·038) | (0·004 to 0·006) | (0·005 to 0·008) | (-0·005 to -0·004) |
| Female=1 | 0·268*** | 0·482*** | 0·160*** | -0·080*** | -0·042*** | -0·035*** | -0·016** | -0·025*** |
|  | (0·200 to 0·336) | (0·429 to 0·535) | (0·130 to 0·189) | (-0·110 to -0·050) | (-0·067 to -0·017) | (-0·046 to -0·023) | (-0·027 to -0·006) | (-0·029 to -0·021) |
| **IMD Deciles - Income (Ref=1)** |  |  |  |  |  |  |  |  |
| 2 | -0·137 | -0·084 | -0·067* | 0·002 | 0·002 | -0·005 | 0·002 | -0·005 |
|  | (-0·306 to 0·032) | (-0·220 to 0·052) | (-0·134 to -0·000) | (-0·076 to 0·079) | (-0·065 to 0·069) | (-0·027 to 0·018) | (-0·017 to 0·022) | (-0·014 to 0·003) |
| 3 | -0·232** | -0·128 | -0·070 | -0·042 | -0·041 | -0·004 | -0·002 | -0·000 |
|  | (-0·406 to -0·058) | (-0·282 to 0·026) | (-0·147 to 0·008) | (-0·106 to 0·022) | (-0·096 to 0·013) | (-0·027 to 0·019) | (-0·022 to 0·018) | (-0·009 to 0·008) |
| 4 | -0·167 | -0·097 | -0·063 | -0·013 | -0·020 | 0·001 | -0·006 | 0·010* |
|  | (-0·347 to 0·013) | (-0·247 to 0·052) | (-0·138 to 0·013) | (-0·085 to 0·059) | (-0·084 to 0·044) | (-0·022 to 0·024) | (-0·026 to 0·013) | (0·002 to 0·019) |
| 5 | -0·076 | -0·007 | -0·049 | -0·006 | -0·012 | 0·001 | -0·001 | 0·006 |
|  | (-0·271 to 0·120) | (-0·174 to 0·160) | (-0·129 to 0·032) | (-0·082 to 0·069) | (-0·080 to 0·055) | (-0·023 to 0·025) | (-0·022 to 0·020) | (-0·002 to 0·015) |
| 6 | -0·092 | -0·007 | -0·074 | 0·013 | -0·004 | 0·012 | 0·005 | 0·012** |
|  | (-0·292 to 0·108) | (-0·175 to 0·161) | (-0·152 to 0·005) | (-0·063 to 0·089) | (-0·070 to 0·062) | (-0·014 to 0·038) | (-0·017 to 0·028) | (0·004 to 0·021) |
| 7 | -0·213* | -0·011 | -0·082 | -0·099** | -0·093** | -0·014 | -0·017 | 0·010* |
|  | (-0·426 to -0·001) | (-0·196 to 0·174) | (-0·169 to 0·004) | (-0·167 to -0·030) | (-0·154 to -0·032) | (-0·037 to 0·010) | (-0·037 to 0·004) | (0·001 to 0·019) |
| 8 | -0·284* | -0·077 | -0·099* | -0·073* | -0·074* | -0·010 | -0·012 | 0·010* |
|  | (-0·515 to -0·053) | (-0·283 to 0·128) | (-0·196 to -0·002) | (-0·146 to -0·000) | (-0·140 to -0·007) | (-0·034 to 0·014) | (-0·034 to 0·009) | (0·001 to 0·019) |
| 9 | -0·292* | -0·086 | -0·101* | -0·068 | -0·106*** | 0·025 | 0·016 | 0·019*** |
|  | (-0·528 to -0·057) | (-0·291 to 0·120) | (-0·196 to -0·007) | (-0·138 to 0·001) | (-0·168 to -0·044) | (-0·001 to 0·051) | (-0·008 to 0·039) | (0·010 to 0·027) |
| 10 = Least | -0·221 | -0·050 | -0·108* | -0·041 | -0·059 | 0·004 | -0·003 | 0·022*** |
|  | (-0·495 to 0·053) | (-0·291 to 0·191) | (-0·209 to -0·006) | (-0·136 to 0·054) | (-0·146 to 0·028) | (-0·023 to 0·031) | (-0·028 to 0·022) | (0·013 to 0·031) |
| **Ethnicity (ref White)** |  |  |  |  |  |  |  |  |
| Asian | 0·025 | 0·250* | 0·061 | -0·111*** | -0·099*** | -0·014 | -0·000 | -0·018*** |
|  | (-0·234 to 0·284) | (0·028 to 0·472) | (-0·033 to 0·155) | (-0·173 to -0·048) | (-0·151 to -0·046) | (-0·037 to 0·009) | (-0·022 to 0·021) | (-0·025 to -0·010) |
| Black | 0·118 | 0·154 | 0·033 | -0·066 | -0·063 | -0·007 | 0·007 | -0·021*** |
|  | (-0·122 to 0·358) | (-0·060 to 0·369) | (-0·072 to 0·138) | (-0·145 to 0·012) | (-0·128 to 0·002) | (-0·035 to 0·021) | (-0·019 to 0·033) | (-0·029 to -0·014) |
| Mixed/Multiple | 0·556*** | 0·489*** | 0·255*** | 0·020 | 0·022 | -0·002 | -0·002 | -0·007 |
|  | (0·297 to 0·815) | (0·295 to 0·684) | (0·133 to 0·377) | (-0·122 to 0·163) | (-0·099 to 0·143) | (-0·042 to 0·038) | (-0·036 to 0·033) | (-0·020 to 0·006) |
| Other | 0·424 | 0·334 | 0·110 | 0·068 | -0·018 | 0·072 | 0·069 | -0·005 |
|  | (-0·285 to 1·133) | (-0·125 to 0·794) | (-0·178 to 0·397) | (-0·340 to 0·476) | (-0·303 to 0·268) | (-0·105 to 0·250) | (-0·099 to 0·237) | (-0·030 to 0·020) |
| unknown | -0·063 | -0·061 | 0·104 | -0·049 | -0·096* | 0·037 | 0·038 | -0·005 |
|  | (-0·536 to 0·411) | (-0·486 to 0·365) | (-0·095 to 0·303) | (-0·162 to 0·064) | (-0·178 to -0·014) | (-0·018 to 0·092) | (-0·012 to 0·089) | (-0·019 to 0·009) |
| **Number of LTCs (ref =0)** |  |  |  |  |  |  |  |  |
| 1 | 0·666*** | 0·737*** | 0·209*** | 0·029 | 0·040 | -0·005 | 0·000 | 0·006** |
|  | (0·557 to 0·775) | (0·653 to 0·822) | (0·162 to 0·256) | (-0·018 to 0·077) | (-0·002 to 0·082) | (-0·018 to 0·007) | (-0·010 to 0·011) | (0·002 to 0·010) |
| 2 | 1·195*** | 1·237*** | 0·344*** | 0·134*** | 0·137*** | 0·009 | 0·011 | 0·018*** |
|  | (1·058 to 1·331) | (1·135 to 1·338) | (0·293 to 0·395) | (0·079 to 0·190) | (0·085 to 0·188) | (-0·007 to 0·025) | (-0·002 to 0·023) | (0·013 to 0·022) |
| 3 | 1·585*** | 1·615*** | 0·427*** | 0·220*** | 0·217*** | 0·020 | 0·021* | 0·025*** |
|  | (1·418 to 1·753) | (1·493 to 1·737) | (0·371 to 0·484) | (0·153 to 0·287) | (0·156 to 0·278) | (-0·002 to 0·041) | (0·003 to 0·038) | (0·020 to 0·030) |
| 4 or more | 2·238*** | 2·357*** | 0·653*** | 0·341*** | 0·304*** | 0·064*** | 0·037*** | 0·087*** |
|  | (2·012 to 2·465) | (2·189 to 2·524) | (0·593 to 0·712) | (0·265 to 0·416) | (0·224 to 0·383) | (0·042 to 0·086) | (0·022 to 0·051) | (0·081 to 0·092) |
| **Region (Ref=North East)** |  |  |  |  |  |  |  |  |
| North West | 0·677 | 0·463 | 0·669*** | -0·052 | -0·021 | -0·027 | -0·024 | 0·002 |
|  | (-0·020 to 1·374) | (-0·161 to 1·087) | (0·407 to 0·931) | (-0·159 to 0·056) | (-0·106 to 0·064) | (-0·066 to 0·012) | (-0·055 to 0·007) | (-0·013 to 0·016) |
| Yorkshire and Humberside | 1·358*** | 0·570* | 0·485*** | 0·224*** | 0·237*** | 0·005 | 0·013 | -0·010 |
|  | (0·763 to 1·953) | (0·011 to 1·129) | (0·278 to 0·692) | (0·124 to 0·325) | (0·152 to 0·323) | (-0·029 to 0·039) | (-0·013 to 0·040) | (-0·022 to 0·003) |
| East Midlands | -0·016 | -0·143 | 0·258* | 0·034 | 0·047 | -0·022 | -0·038** | 0·023** |
|  | (-0·700 to 0·669) | (-0·785 to 0·499) | (0·019 to 0·497) | (-0·090 to 0·158) | (-0·065 to 0·158) | (-0·059 to 0·014) | (-0·065 to -0·011) | (0·008 to 0·038) |
| West Midlands | 0·596 | 0·202 | 0·291** | 0·104 | 0·096* | 0·013 | 0·015 | 0·001 |
|  | (-0·006 to 1·199) | (-0·358 to 0·763) | (0·094 to 0·488) | (-0·006 to 0·214) | (0·001 to 0·190) | (-0·022 to 0·048) | (-0·013 to 0·043) | (-0·011 to 0·012) |
| East of England | 0·928** | 0·375 | 0·304** | 0·255*** | 0·272*** | 0·003 | -0·003 | 0·009 |
|  | (0·339 to 1·517) | (-0·170 to 0·919) | (0·113 to 0·495) | (0·156 to 0·354) | (0·188 to 0·355) | (-0·030 to 0·036) | (-0·028 to 0·022) | (-0·003 to 0·020) |
| London | 0·679* | 0·660* | 0·420*** | -0·027 | -0·022 | -0·004 | 0·002 | -0·003 |
|  | (0·075 to 1·282) | (0·095 to 1·225) | (0·201 to 0·640) | (-0·113 to 0·059) | (-0·089 to 0·044) | (-0·038 to 0·029) | (-0·024 to 0·027) | (-0·015 to 0·009) |
| South East | 0·792** | 0·317 | 0·296** | 0·122** | 0·143*** | -0·002 | -0·010 | 0·009 |
|  | (0·219 to 1·364) | (-0·221 to 0·856) | (0·101 to 0·490) | (0·034 to 0·210) | (0·071 to 0·215) | (-0·035 to 0·031) | (-0·035 to 0·015) | (-0·003 to 0·021) |
| South West | -0·106 | -0·164 | 0·153 | -0·033 | -0·015 | -0·007 | -0·011 | 0·002 |
|  | (-0·877 to 0·665) | (-0·856 to 0·528) | (-0·107 to 0·412) | (-0·156 to 0·090) | (-0·120 to 0·090) | (-0·046 to 0·031) | (-0·041 to 0·019) | (-0·013 to 0·017) |
| **Referral Year (ref=2019)** |  |  |  |  |  |  |  |  |
| 2020 | 0·183 | 0·210* | 0·288*** | 0·028 | 0·032 | -0·008 | -0·000 | -0·003 |
|  | (-0·018 to 0·384) | (0·045 to 0·375) | (0·199 to 0·377) | (-0·054 to 0·110) | (-0·042 to 0·105) | (-0·033 to 0·017) | (-0·022 to 0·021) | (-0·015 to 0·008) |
| 2021 | 0·595*** | 0·461*** | 0·279*** | 0·435*** | 0·361*** | 0·070*** | 0·055*** | 0·012* |
|  | (0·386 to 0·803) | (0·289 to 0·633) | (0·188 to 0·371) | (0·355 to 0·515) | (0·290 to 0·432) | (0·046 to 0·094) | (0·035 to 0·076) | (0·001 to 0·023) |
| 2022 | 0·208 | 0·101 | -0·015 | 0·568*** | 0·455*** | 0·108*** | 0·096*** | 0·005 |
|  | (-0·161 to 0·577) | (-0·220 to 0·422) | (-0·170 to 0·141) | (0·465 to 0·671) | (0·365 to 0·544) | (0·076 to 0·140) | (0·069 to 0·123) | (-0·009 to 0·019) |
| **Referral Month (ref=Jan)** |  |  |  |  |  |  |  |  |
| Feb | -0·573** | -0·485** | -0·223** | -0·082 | -0·073 | -0·012 | -0·010 | -0·002 |
|  | (-0·925 to -0·222) | (-0·780 to -0·191) | (-0·358 to -0·089) | (-0·169 to 0·006) | (-0·145 to 0·000) | (-0·037 to 0·013) | (-0·031 to 0·010) | (-0·013 to 0·009) |
| Mar | -0·537*** | -0·399*** | -0·245*** | -0·118** | -0·097** | -0·021 | -0·014 | -0·009* |
|  | (-0·756 to -0·318) | (-0·587 to -0·211) | (-0·330 to -0·160) | (-0·189 to -0·047) | (-0·157 to -0·037) | (-0·044 to 0·001) | (-0·035 to 0·006) | (-0·017 to -0·001) |
| Apr | -0·576*** | -0·431*** | -0·303*** | -0·155*** | -0·108** | -0·049*** | -0·028* | -0·020*** |
|  | (-0·829 to -0·322) | (-0·652 to -0·210) | (-0·412 to -0·193) | (-0·239 to -0·071) | (-0·181 to -0·036) | (-0·077 to -0·021) | (-0·053 to -0·003) | (-0·030 to -0·011) |
| May | -0·203 | -0·196 | -0·223*** | -0·002 | -0·005 | 0·001 | 0·007 | -0·009 |
|  | (-0·475 to 0·069) | (-0·429 to 0·037) | (-0·338 to -0·109) | (-0·085 to 0·081) | (-0·077 to 0·066) | (-0·028 to 0·030) | (-0·019 to 0·034) | (-0·019 to 0·001) |
| Jun | 0·155 | 0·114 | -0·090 | 0·100* | 0·070* | 0·027 | 0·033* | -0·006 |
|  | (-0·099 to 0·410) | (-0·107 to 0·335) | (-0·199 to 0·020) | (0·017 to 0·183) | (0·001 to 0·140) | (-0·005 to 0·059) | (0·003 to 0·062) | (-0·016 to 0·004) |
| Jul | 0·043 | 0·011 | -0·170** | 0·122** | 0·073* | 0·045* | 0·038* | 0·004 |
|  | (-0·207 to 0·294) | (-0·206 to 0·228) | (-0·279 to -0·061) | (0·037 to 0·208) | (0·001 to 0·144) | (0·010 to 0·081) | (0·004 to 0·072) | (-0·006 to 0·014) |
| Aug | 0·243 | 0·237* | -0·020 | 0·140** | 0·102* | 0·035* | 0·025 | 0·008 |
|  | (-0·013 to 0·498) | (0·023 to 0·452) | (-0·129 to 0·088) | (0·050 to 0·231) | (0·024 to 0·181) | (0·005 to 0·066) | (-0·002 to 0·052) | (-0·003 to 0·019) |
| Sep | 0·159 | 0·182 | -0·017 | 0·106* | 0·085* | 0·018 | 0·027* | -0·009 |
|  | (-0·082 to 0·400) | (-0·026 to 0·390) | (-0·119 to 0·085) | (0·022 to 0·189) | (0·013 to 0·158) | (-0·009 to 0·045) | (0·003 to 0·051) | (-0·019 to 0·001) |
| Oct | 0·165 | 0·126 | -0·030 | 0·129** | 0·073 | 0·049** | 0·047** | 0·002 |
|  | (-0·113 to 0·443) | (-0·121 to 0·372) | (-0·150 to 0·090) | (0·035 to 0·222) | (-0·017 to 0·163) | (0·017 to 0·080) | (0·017 to 0·077) | (-0·008 to 0·012) |
| Nov | -0·272* | -0·199 | -0·146** | 0·080* | 0·060* | 0·017 | 0·018 | -0·004 |
|  | (-0·512 to -0·031) | (-0·414 to 0·016) | (-0·246 to -0·046) | (0·006 to 0·153) | (0·001 to 0·120) | (-0·011 to 0·045) | (-0·007 to 0·043) | (-0·014 to 0·005) |
| Dec | -0·452** | -0·348** | -0·231*** | 0·070 | 0·045 | 0·022 | 0·021 | -0·001 |
|  | (-0·730 to -0·173) | (-0·591 to -0·104) | (-0·345 to -0·117) | (-0·013 to 0·153) | (-0·025 to 0·115) | (-0·005 to 0·049) | (-0·004 to 0·045) | (-0·010 to 0·009) |
| Constant | 0·216 | 1·618*** | 0·909*** | -1·144*** | -0·963*** | -0·178*** | -0·192*** | 0·073*** |
|  | (-0·600 to 1·031) | (0·878 to 2·358) | (0·600 to 1·218) | (-1·337 to -0·952) | (-1·132 to -0·795) | (-0·238 to -0·118) | (-0·242 to -0·142) | (0·049 to 0·097) |

**Note:** N= 1,471,142, 95% Confidence Intervals based on clustered standard errors at general practice (N=1,432) level in parentheses. * p<0·05, ** p<0·01, *** p<0·001.

**Supplementary Table 17: Coefficients from Models in Table 4 – healthcare use – 1 year follow-up**

|  | (1) | (2) | (3) | (4) | (5) | (6) | (7) | (8) |
| --- | --- | --- | --- | --- | --- | --- | --- | --- |
|  | Primary Care and Hospital | All primary care | General Practitioner | All Hospital | Outpatient | Admitted Patient Care | Admitted Patient Care - Elective | Admitted Patient Care - Emergency |
| 2 year Lag of Outcome | 0·306*** | 0·188** | -0·048** | -0·022 | -0·026 | 0·005 | -0·001 | 0·006** |
|  | (0·147 to 0·464) | (0·050 to 0·326) | (-0·084 to -0·012) | (-0·053 to 0·009) | (-0·052 to 0·000) | (-0·005 to 0·015) | (-0·009 to 0·007) | (0·002 to 0·010) |
| Age | 0·315*** | 0·234*** | 0·545*** | 0·537*** | 0·539*** | 0·525*** | 0·523*** | 0·556*** |
|  | (0·302 to 0·327) | (0·223 to 0·246) | (0·542 to 0·548) | (0·528 to 0·545) | (0·527 to 0·552) | (0·517 to 0·533) | (0·515 to 0·532) | (0·550 to 0·561) |
| Age^2 | -0·021** | -0·073*** | -0·026*** | 0·021*** | 0·016*** | 0·005*** | 0·004*** | 0·001*** |
|  | (-0·034 to -0·008) | (-0·085 to -0·062) | (-0·030 to -0·023) | (0·017 to 0·024) | (0·013 to 0·019) | (0·003 to 0·006) | (0·003 to 0·005) | (0·001 to 0·002) |
| Female=1 | 0·130** | 0·597*** | -0·124*** | -0·094*** | -0·059*** | -0·038*** | -0·017*** | -0·016*** |
|  | (0·048 to 0·212) | (0·530 to 0·664) | (-0·146 to -0·102) | (-0·119 to -0·069) | (-0·080 to -0·038) | (-0·047 to -0·029) | (-0·025 to -0·008) | (-0·020 to -0·013) |
| **IMD Deciles - Income (Ref=1)** |  |  |  |  |  |  |  |  |
| 2 | -0·190 | -0·070 | -0·002 | 0·041 | 0·041 | 0·001 | 0·001 | -0·001 |
|  | (-0·428 to 0·049) | (-0·252 to 0·112) | (-0·061 to 0·056) | (-0·012 to 0·093) | (-0·005 to 0·086) | (-0·016 to 0·018) | (-0·014 to 0·016) | (-0·007 to 0·006) |
| 3 | -0·313** | -0·141 | -0·023 | 0·010 | 0·010 | 0·000 | -0·002 | 0·000 |
|  | (-0·547 to -0·079) | (-0·328 to 0·046) | (-0·080 to 0·035) | (-0·045 to 0·065) | (-0·035 to 0·056) | (-0·018 to 0·019) | (-0·019 to 0·014) | (-0·006 to 0·007) |
| 4 | -0·271* | -0·105 | -0·002 | -0·002 | -0·003 | 0·003 | -0·004 | 0·003 |
|  | (-0·518 to -0·025) | (-0·295 to 0·085) | (-0·059 to 0·054) | (-0·059 to 0·054) | (-0·051 to 0·045) | (-0·017 to 0·023) | (-0·022 to 0·013) | (-0·004 to 0·010) |
| 5 | -0·165 | 0·010 | 0·002 | 0·009 | 0·014 | -0·004 | -0·007 | 0·001 |
|  | (-0·435 to 0·104) | (-0·206 to 0·227) | (-0·060 to 0·063) | (-0·046 to 0·064) | (-0·033 to 0·060) | (-0·022 to 0·015) | (-0·024 to 0·009) | (-0·006 to 0·008) |
| 6 | -0·245 | 0·018 | -0·003 | 0·017 | 0·011 | 0·007 | 0·007 | -0·002 |
|  | (-0·546 to 0·057) | (-0·215 to 0·251) | (-0·069 to 0·063) | (-0·041 to 0·075) | (-0·038 to 0·061) | (-0·016 to 0·030) | (-0·015 to 0·029) | (-0·009 to 0·005) |
| 7 | -0·312* | -0·020 | -0·052 | 0·008 | 0·011 | -0·001 | -0·006 | 0·001 |
|  | (-0·611 to -0·013) | (-0·257 to 0·216) | (-0·117 to 0·013) | (-0·047 to 0·062) | (-0·034 to 0·057) | (-0·021 to 0·019) | (-0·024 to 0·012) | (-0·007 to 0·008) |
| 8 | -0·394* | -0·018 | -0·041 | 0·015 | 0·008 | 0·009 | 0·006 | -0·002 |
|  | (-0·695 to -0·093) | (-0·267 to 0·231) | (-0·107 to 0·025) | (-0·042 to 0·073) | (-0·042 to 0·059) | (-0·010 to 0·029) | (-0·012 to 0·024) | (-0·009 to 0·005) |
| 9 | -0·383* | 0·029 | -0·029 | -0·005 | -0·003 | 0·002 | 0·004 | -0·004 |
|  | (-0·714 to -0·053) | (-0·239 to 0·296) | (-0·097 to 0·039) | (-0·061 to 0·051) | (-0·053 to 0·046) | (-0·018 to 0·021) | (-0·014 to 0·021) | (-0·012 to 0·003) |
| 10 = Least | -0·415* | -0·012 | -0·058 | -0·019 | -0·007 | -0·008 | -0·007 | -0·007 |
|  | (-0·789 to -0·042) | (-0·315 to 0·291) | (-0·128 to 0·012) | (-0·076 to 0·038) | (-0·058 to 0·044) | (-0·028 to 0·011) | (-0·025 to 0·011) | (-0·014 to 0·001) |
| **Ethnicity (ref White)** |  |  |  |  |  |  |  |  |
| Asian | -0·056 | 0·291 | -0·080* | -0·027 | -0·016 | -0·010 | -0·006 | -0·005 |
|  | (-0·442 to 0·331) | (-0·007 to 0·589) | (-0·150 to -0·010) | (-0·074 to 0·021) | (-0·055 to 0·022) | (-0·031 to 0·010) | (-0·024 to 0·013) | (-0·010 to 0·001) |
| Black | 0·117 | 0·089 | 0·023 | -0·060 | -0·031 | -0·026 | -0·023 | 0·001 |
|  | (-0·168 to 0·401) | (-0·174 to 0·352) | (-0·056 to 0·103) | (-0·132 to 0·012) | (-0·086 to 0·023) | (-0·057 to 0·005) | (-0·051 to 0·005) | (-0·007 to 0·008) |
| Mixed/Multiple | 0·512*** | 0·523*** | 0·127** | -0·157** | -0·106* | -0·049* | -0·040* | -0·001 |
|  | (0·236 to 0·788) | (0·307 to 0·739) | (0·032 to 0·223) | (-0·276 to -0·039) | (-0·202 to -0·010) | (-0·090 to -0·007) | (-0·078 to -0·003) | (-0·013 to 0·011) |
| Other | 1·072*** | 0·741*** | 0·239* | 0·287 | 0·124 | 0·168 | 0·156 | 0·008 |
|  | (0·481 to 1·662) | (0·302 to 1·179) | (0·021 to 0·457) | (-0·015 to 0·589) | (-0·056 to 0·303) | (-0·059 to 0·395) | (-0·070 to 0·382) | (-0·013 to 0·029) |
| unknown | 0·191 | -0·018 | 0·204*** | 0·108* | 0·103** | 0·008 | -0·007 | 0·009 |
|  | (-0·241 to 0·623) | (-0·441 to 0·405) | (0·105 to 0·303) | (0·021 to 0·194) | (0·030 to 0·175) | (-0·023 to 0·039) | (-0·034 to 0·021) | (-0·003 to 0·021) |
| **Number of LTCs (ref =0)** |  |  |  |  |  |  |  |  |
| 1 | 1·029*** | 1·278*** | 0·093*** | -0·011 | -0·012 | 0·001 | 0·006 | -0·002 |
|  | (0·906 to 1·152) | (1·180 to 1·376) | (0·060 to 0·126) | (-0·044 to 0·022) | (-0·042 to 0·017) | (-0·008 to 0·010) | (-0·002 to 0·013) | (-0·005 to 0·002) |
| 2 | 1·459*** | 1·891*** | 0·024 | -0·024 | -0·029 | 0·005 | 0·008 | 0·001 |
|  | (1·268 to 1·649) | (1·746 to 2·035) | (-0·013 to 0·062) | (-0·065 to 0·016) | (-0·069 to 0·010) | (-0·005 to 0·016) | (-0·000 to 0·017) | (-0·003 to 0·005) |
| 3 | 1·749*** | 2·366*** | -0·039 | -0·050* | -0·051* | 0·002 | 0·009 | -0·006** |
|  | (1·482 to 2·016) | (2·165 to 2·567) | (-0·080 to 0·003) | (-0·098 to -0·002) | (-0·100 to -0·003) | (-0·012 to 0·016) | (-0·004 to 0·021) | (-0·010 to -0·001) |
| 4 or more | 2·196*** | 3·352*** | -0·091*** | -0·130*** | -0·132*** | 0·007 | 0·003 | -0·007** |
|  | (1·747 to 2·644) | (3·012 to 3·692) | (-0·133 to -0·050) | (-0·199 to -0·062) | (-0·208 to -0·056) | (-0·008 to 0·023) | (-0·008 to 0·014) | (-0·011 to -0·002) |
| **Region (Ref=North East)** |  |  |  |  |  |  |  |  |
| North West | 1·263** | 0·649 | 0·251** | -0·137* | -0·104 | -0·032 | -0·030 | -0·001 |
|  | (0·332 to 2·193) | (-0·045 to 1·344) | (0·064 to 0·439) | (-0·265 to -0·010) | (-0·211 to 0·003) | (-0·072 to 0·009) | (-0·062 to 0·002) | (-0·015 to 0·012) |
| Yorkshire and Humberside | 2·800*** | 1·316*** | 0·179** | -0·105* | -0·092* | -0·015 | -0·004 | -0·010 |
|  | (2·133 to 3·467) | (0·803 to 1·829) | (0·043 to 0·315) | (-0·198 to -0·013) | (-0·177 to -0·007) | (-0·049 to 0·018) | (-0·033 to 0·024) | (-0·021 to 0·001) |
| East Midlands | -0·291 | -0·607 | 0·048 | 0·071 | 0·063 | 0·013 | 0·000 | 0·009 |
|  | (-1·228 to 0·645) | (-1·322 to 0·107) | (-0·099 to 0·194) | (-0·022 to 0·164) | (-0·022 to 0·147) | (-0·021 to 0·047) | (-0·029 to 0·029) | (-0·004 to 0·023) |
| West Midlands | 0·946** | 0·141 | 0·074 | -0·005 | -0·014 | 0·010 | 0·006 | 0·006 |
|  | (0·228 to 1·663) | (-0·393 to 0·676) | (-0·056 to 0·205) | (-0·087 to 0·077) | (-0·087 to 0·059) | (-0·022 to 0·041) | (-0·020 to 0·031) | (-0·005 to 0·018) |
| East of England | 1·604*** | 0·679** | 0·114 | 0·056 | 0·037 | 0·016 | 0·008 | 0·008 |
|  | (0·919 to 2·289) | (0·195 to 1·162) | (-0·019 to 0·246) | (-0·027 to 0·139) | (-0·038 to 0·112) | (-0·015 to 0·046) | (-0·018 to 0·034) | (-0·003 to 0·019) |
| London | 0·832* | 0·717* | 0·064 | -0·018 | -0·008 | -0·009 | -0·006 | -0·001 |
|  | (0·085 to 1·578) | (0·148 to 1·285) | (-0·076 to 0·204) | (-0·101 to 0·064) | (-0·080 to 0·064) | (-0·041 to 0·023) | (-0·032 to 0·020) | (-0·013 to 0·011) |
| South East | 1·143*** | 0·150 | 0·123 | -0·011 | -0·016 | 0·001 | -0·004 | 0·007 |
|  | (0·487 to 1·798) | (-0·325 to 0·624) | (-0·006 to 0·253) | (-0·093 to 0·072) | (-0·093 to 0·060) | (-0·029 to 0·031) | (-0·029 to 0·021) | (-0·004 to 0·018) |
| South West | 0·017 | -0·232 | 0·013 | -0·030 | -0·039 | 0·007 | 0·016 | -0·007 |
|  | (-1·308 to 1·341) | (-1·166 to 0·702) | (-0·148 to 0·174) | (-0·133 to 0·074) | (-0·131 to 0·052) | (-0·027 to 0·042) | (-0·013 to 0·046) | (-0·020 to 0·007) |
| **Referral Year (ref=2019)** |  |  |  |  |  |  |  |  |
| 2020 | -0·598*** | -0·363*** | 0·098** | -0·104** | -0·075** | -0·026* | -0·014 | -0·017*** |
|  | (-0·807 to -0·389) | (-0·528 to -0·199) | (0·027 to 0·168) | (-0·172 to -0·037) | (-0·131 to -0·019) | (-0·050 to -0·002) | (-0·035 to 0·008) | (-0·027 to -0·008) |
| 2021 | -0·766*** | -0·137 | 0·403*** | 0·199*** | 0·190*** | 0·009 | 0·003 | 0·002 |
|  | (-0·996 to -0·535) | (-0·337 to 0·062) | (0·329 to 0·478) | (0·136 to 0·261) | (0·138 to 0·241) | (-0·014 to 0·032) | (-0·017 to 0·024) | (-0·007 to 0·011) |
| 2022 | -0·688*** | -0·234 | 0·430*** | 0·820*** | 0·687*** | 0·133*** | 0·098*** | 0·026*** |
|  | (-1·062 to -0·314) | (-0·584 to 0·115) | (0·328 to 0·532) | (0·741 to 0·898) | (0·622 to 0·751) | (0·105 to 0·160) | (0·074 to 0·122) | (0·016 to 0·037) |
| **Referral Month (ref=Jan)** |  |  |  |  |  |  |  |  |
| Feb | -0·626*** | -0·581*** | -0·124** | -0·019 | -0·023 | 0·005 | 0·011 | -0·006 |
|  | (-0·952 to -0·299) | (-0·877 to -0·285) | (-0·201 to -0·047) | (-0·071 to 0·034) | (-0·067 to 0·022) | (-0·013 to 0·022) | (-0·003 to 0·026) | (-0·014 to 0·002) |
| Mar | -0·232* | -0·148 | -0·082* | -0·023 | -0·019 | -0·005 | 0·004 | -0·008* |
|  | (-0·458 to -0·006) | (-0·359 to 0·062) | (-0·149 to -0·016) | (-0·080 to 0·034) | (-0·067 to 0·028) | (-0·022 to 0·013) | (-0·011 to 0·019) | (-0·014 to -0·001) |
| Apr | -0·056 | -0·317** | -0·094* | 0·226*** | 0·168*** | 0·060*** | 0·057*** | 0·003 |
|  | (-0·306 to 0·193) | (-0·544 to -0·089) | (-0·176 to -0·012) | (0·158 to 0·293) | (0·111 to 0·224) | (0·036 to 0·083) | (0·037 to 0·076) | (-0·005 to 0·011) |
| May | 0·021 | -0·240* | 0·017 | 0·273*** | 0·226*** | 0·049*** | 0·046*** | 0·003 |
|  | (-0·244 to 0·286) | (-0·476 to -0·005) | (-0·061 to 0·095) | (0·203 to 0·342) | (0·168 to 0·283) | (0·027 to 0·071) | (0·026 to 0·066) | (-0·005 to 0·012) |
| Jun | 0·173 | 0·044 | 0·099* | 0·292*** | 0·225*** | 0·068*** | 0·061*** | 0·006 |
|  | (-0·084 to 0·429) | (-0·193 to 0·281) | (0·021 to 0·178) | (0·229 to 0·355) | (0·173 to 0·276) | (0·042 to 0·094) | (0·037 to 0·084) | (-0·002 to 0·015) |
| Jul | 0·243 | 0·150 | 0·146*** | 0·346*** | 0·265*** | 0·083*** | 0·063*** | 0·018*** |
|  | (-0·005 to 0·492) | (-0·074 to 0·374) | (0·073 to 0·219) | (0·266 to 0·426) | (0·191 to 0·339) | (0·055 to 0·111) | (0·038 to 0·089) | (0·010 to 0·027) |
| Aug | 0·152 | 0·158 | 0·112** | 0·377*** | 0·304*** | 0·072*** | 0·055*** | 0·016*** |
|  | (-0·097 to 0·400) | (-0·072 to 0·388) | (0·038 to 0·186) | (0·312 to 0·442) | (0·249 to 0·359) | (0·051 to 0·093) | (0·037 to 0·072) | (0·008 to 0·025) |
| Sep | 0·013 | -0·022 | 0·087* | 0·402*** | 0·317*** | 0·084*** | 0·062*** | 0·017*** |
|  | (-0·219 to 0·244) | (-0·239 to 0·194) | (0·008 to 0·167) | (0·339 to 0·464) | (0·264 to 0·370) | (0·063 to 0·105) | (0·045 to 0·080) | (0·009 to 0·026) |
| Oct | 0·229 | 0·178 | 0·135*** | 0·289*** | 0·242*** | 0·051*** | 0·039*** | 0·011* |
|  | (-0·035 to 0·493) | (-0·041 to 0·397) | (0·061 to 0·208) | (0·194 to 0·384) | (0·164 to 0·319) | (0·027 to 0·075) | (0·021 to 0·058) | (0·002 to 0·021) |
| Nov | -0·199 | -0·112 | 0·111** | 0·343*** | 0·279*** | 0·064*** | 0·049*** | 0·013** |
|  | (-0·438 to 0·041) | (-0·341 to 0·116) | (0·041 to 0·181) | (0·277 to 0·409) | (0·222 to 0·336) | (0·044 to 0·084) | (0·033 to 0·065) | (0·005 to 0·021) |
| Dec | -0·180 | -0·114 | 0·097* | 0·425*** | 0·349*** | 0·075*** | 0·061*** | 0·011** |
|  | (-0·450 to 0·091) | (-0·376 to 0·148) | (0·013 to 0·181) | (0·361 to 0·488) | (0·296 to 0·402) | (0·054 to 0·097) | (0·043 to 0·079) | (0·003 to 0·020) |
| Constant | 3·243*** | 4·772*** | 0·972*** | -0·830*** | -0·673*** | -0·160*** | -0·136*** | -0·036*** |
|  | (2·388 to 4·098) | (4·100 to 5·444) | (0·772 to 1·173) | (-0·980 to -0·680) | (-0·802 to -0·543) | (-0·209 to -0·111) | (-0·177 to -0·094) | (-0·057 to -0·016) |

**Note:** N=1,471,142, 95% Confidence Intervals based on clustered standard errors at general practice (N=1,432) level in parentheses. * p<0·05, ** p<0·01, *** p<0·001.

**Supplementary Table 18: Coefficients from Models in Table 4 – healthcare cost – 6 months follow-up**

|  | (1) | (2) | (3) | (4) | (5) | (6) | (7) | (8) |
| --- | --- | --- | --- | --- | --- | --- | --- | --- |
|  | Primary Care and Hospital | All primary care | General Practitioner | All Hospital | Outpatient | Admitted Patient Care | Admitted Patient Care - Emergency | Admitted Patient Care - Elective |
| 2 year Lag of Outcome | 0·287*** | 0·202*** | 0·269*** | 0·287*** | 0·280*** | 0·289*** | 0·235*** | 0·313*** |
|  | (0·279 to 0·296) | (0·197 to 0·208) | (0·267 to 0·271) | (0·279 to 0·296) | (0·274 to 0·287) | (0·278 to 0·299) | (0·210 to 0·260) | (0·305 to 0·321) |
| Age | 3·972*** | -1·618*** | -1·184*** | 3·972*** | 2·535*** | 1·651 | 4·164*** | 1·632 |
|  | (2·125 to 5·819) | (-1·849 to -1·387) | (-1·355 to -1·012) | (2·125 to 5·818) | (2·224 to 2·846) | (-0·146 to 3·448) | (2·788 to 5·540) | (-0·032 to 3·296) |
| Age^2 | -0·011 | 0·008*** | 0·008*** | -0·010 | -0·022*** | 0·010 | -0·034*** | 0·004 |
|  | (-0·028 to 0·006) | (0·006 to 0·010) | (0·007 to 0·009) | (-0·028 to 0·007) | (-0·025 to -0·020) | (-0·007 to 0·027) | (-0·045 to -0·022) | (-0·011 to 0·020) |
| Female=1 | -73·423*** | 2·925*** | -3·138*** | -73·167*** | -5·174*** | -67·944*** | -16·027*** | -48·512*** |
|  | (-88·337 to -58·510) | (1·606 to 4·245) | (-4·053 to -2·223) | (-88·086 to -58·248) | (-7·974 to -2·374) | (-82·231 to -53·656) | (-23·510 to -8·543) | (-59·938 to -37·086) |
| **IMD Deciles - Income (Ref=1)** |  |  |  |  |  |  |  |  |
| 2 | -10·899 | -2·214 | -0·625 | -10·700 | -1·293 | -9·844 | 10·177 | -22·114 |
|  | (-40·854 to 19·055) | (-5·551 to 1·124) | (-2·916 to 1·667) | (-40·649 to 19·249) | (-7·623 to 5·038) | (-38·230 to 18·543) | (-2·387 to 22·742) | (-46·453 to 2·225) |
| 3 | -23·482 | -3·662* | -1·175 | -23·217 | -1·377 | -22·337 | 2·768 | -27·738* |
|  | (-58·098 to 11·134) | (-7·148 to -0·177) | (-3·573 to 1·223) | (-57·849 to 11·415) | (-7·770 to 5·017) | (-55·226 to 10·551) | (-12·516 to 18·052) | (-54·625 to -0·851) |
| 4 | 7·632 | -1·761 | 0·203 | 7·866 | -1·690 | 8·961 | 13·333 | -8·363 |
|  | (-23·836 to 39·100) | (-5·293 to 1·772) | (-2·202 to 2·607) | (-23·607 to 39·338) | (-8·260 to 4·881) | (-20·825 to 38·747) | (-1·237 to 27·903) | (-34·086 to 17·359) |
| 5 | -9·932 | -2·853 | -1·483 | -9·815 | -4·208 | -6·379 | -8·909 | -6·257 |
|  | (-42·948 to 23·085) | (-6·612 to 0·906) | (-3·971 to 1·005) | (-42·850 to 23·220) | (-10·532 to 2·115) | (-37·787 to 25·028) | (-21·095 to 3·276) | (-33·786 to 21·273) |
| 6 | 8·933 | -3·130 | -0·974 | 9·256 | 1·392 | 7·059 | 13·162 | -13·869 |
|  | (-22·904 to 40·770) | (-7·241 to 0·980) | (-3·576 to 1·627) | (-22·572 to 41·083) | (-5·412 to 8·196) | (-22·858 to 36·976) | (-0·468 to 26·793) | (-39·662 to 11·923) |
| 7 | 8·983 | -4·051 | -2·259 | 9·290 | 0·580 | 7·676 | -1·507 | -1·848 |
|  | (-23·220 to 41·186) | (-8·154 to 0·053) | (-4·982 to 0·465) | (-22·927 to 41·507) | (-5·951 to 7·112) | (-22·561 to 37·913) | (-14·569 to 11·556) | (-28·039 to 24·344) |
| 8 | 14·154 | -4·792* | -3·133* | 14·532 | -1·915 | 15·122 | 2·931 | -1·097 |
|  | (-18·250 to 46·558) | (-8·962 to -0·622) | (-5·932 to -0·334) | (-17·890 to 46·955) | (-8·047 to 4·216) | (-15·667 to 45·911) | (-10·527 to 16·388) | (-27·601 to 25·408) |
| 9 | 6·413 | -3·318 | -2·159 | 6·769 | -2·077 | 7·418 | 5·467 | -14·183 |
|  | (-26·823 to 39·649) | (-7·951 to 1·315) | (-4·994 to 0·676) | (-26·469 to 40·007) | (-8·359 to 4·205) | (-23·993 to 38·828) | (-9·106 to 20·040) | (-40·633 to 12·267) |
| 10 = Least | -10·374 | -4·353 | -3·404* | -10·138 | -5·342 | -6·325 | -7·315 | -19·299 |
|  | (-44·103 to 23·356) | (-9·451 to 0·745) | (-6·471 to -0·337) | (-43·887 to 23·612) | (-12·080 to 1·395) | (-37·822 to 25·172) | (-22·442 to 7·813) | (-45·681 to 7·083) |
| **Ethnicity (ref White)** |  |  |  |  |  |  |  |  |
| Asian | -0·050 | 0·148 | -3·989* | 0·267 | -4·881* | 5·203 | 4·761 | -4·026 |
|  | (-22·318 to 22·219) | (-5·833 to 6·129) | (-7·117 to -0·861) | (-22·014 to 22·548) | (-9·643 to -0·119) | (-15·626 to 26·033) | (-6·079 to 15·602) | (-21·684 to 13·633) |
| Black | -16·377 | 1·968 | 0·885 | -16·527 | -2·268 | -14·031 | 2·419 | -17·346 |
|  | (-46·881 to 14·128) | (-3·193 to 7·130) | (-2·639 to 4·409) | (-47·037 to 13·983) | (-8·453 to 3·918) | (-42·222 to 14·160) | (-12·593 to 17·430) | (-41·208 to 6·516) |
| Mixed/Multiple | -20·773 | 10·527*** | 5·279** | -21·137 | -1·707 | -19·226 | -16·643 | 1·552 |
|  | (-65·104 to 23·559) | (5·940 to 15·115) | (1·589 to 8·969) | (-65·462 to 23·187) | (-11·048 to 7·635) | (-60·706 to 22·254) | (-38·366 to 5·080) | (-31·416 to 34·520) |
| Other | -7·556 | 18·731*** | 11·208* | -8·239 | 15·293 | -23·757 | 21·975 | -51·919 |
|  | (-93·412 to 78·301) | (7·896 to 29·566) | (1·998 to 20·417) | (-94·111 to 77·633) | (-10·169 to 40·755) | (-100·335 to 52·822) | (-15·080 to 59·030) | (-126·190 to 22·352) |
| unknown | 26·101 | 7·515 | 7·112* | 25·409 | 8·019 | 16·974 | -4·457 | 13·054 |
|  | (-20·863 to 73·064) | (-0·691 to 15·721) | (1·587 to 12·638) | (-21·425 to 72·243) | (-4·831 to 20·870) | (-26·856 to 60·804) | (-21·326 to 12·411) | (-27·990 to 54·099) |
| **Number of LTCs (ref =0)** |  |  |  |  |  |  |  |  |
| 1 | 4·111 | 15·240*** | 6·320*** | 4·590 | 0·365 | 4·788 | 3·616 | 2·567 |
|  | (-11·223 to 19·446) | (13·276 to 17·204) | (4·866 to 7·774) | (-10·732 to 19·912) | (-2·592 to 3·322) | (-9·506 to 19·083) | (-4·810 to 12·043) | (-7·849 to 12·983) |
| 2 | 20·379* | 17·203*** | 4·824*** | 21·433* | 4·246* | 18·307* | 17·022** | 6·030 |
|  | (1·999 to 38·759) | (14·631 to 19·775) | (3·234 to 6·413) | (3·090 to 39·775) | (0·838 to 7·655) | (1·536 to 35·077) | (6·371 to 27·673) | (-5·519 to 17·579) |
| 3 | 1·319 | 16·016*** | 2·125* | 3·098 | -0·072 | 4·892 | 16·586* | -5·523 |
|  | (-19·836 to 22·473) | (12·887 to 19·145) | (0·390 to 3·859) | (-17·989 to 24·185) | (-4·248 to 4·104) | (-14·696 to 24·479) | (3·665 to 29·506) | (-19·154 to 8·107) |
| 4 or more | 6·061 | 16·972*** | 2·252* | 10·144 | -8·034** | 20·590 | 11·741 | 0·570 |
|  | (-25·763 to 37·885) | (12·656 to 21·289) | (0·489 to 4·015) | (-21·468 to 41·756) | (-12·843 to -3·225) | (-9·008 to 50·189) | (-7·226 to 30·708) | (-16·428 to 17·568) |
| **Region (Ref=North East)** |  |  |  |  |  |  |  |  |
| North West | 27·665 | 23·090*** | 12·258*** | 26·047 | 0·394 | 26·859 | 8·194 | 17·438 |
|  | (-48·055 to 103·385) | (9·491 to 36·688) | (5·554 to 18·961) | (-49·854 to 101·947) | (-10·988 to 11·776) | (-42·441 to 96·159) | (-19·888 to 36·275) | (-37·902 to 72·778) |
| Yorkshire and Humberside | -29·633 | 28·392*** | 8·577** | -32·592 | 0·585 | -31·040 | 3·066 | -28·926 |
|  | (-89·031 to 29·766) | (16·416 to 40·367) | (2·793 to 14·361) | (-91·914 to 26·731) | (-7·968 to 9·138) | (-86·404 to 24·324) | (-23·391 to 29·523) | (-73·574 to 15·723) |
| East Midlands | 37·793 | -0·707 | 4·053 | 37·208 | 21·787*** | 14·318 | 14·089 | 0·346 |
|  | (-24·551 to 100·138) | (-13·998 to 12·584) | (-2·039 to 10·145) | (-25·048 to 99·464) | (9·559 to 34·015) | (-45·302 to 73·938) | (-14·708 to 42·886) | (-48·163 to 48·855) |
| West Midlands | 66·153* | 8·940 | 4·573 | 64·539* | 11·035** | 54·605 | 28·749* | 25·193 |
|  | (5·800 to 126·506) | (-3·115 to 20·994) | (-0·875 to 10·020) | (4·248 to 124·829) | (2·693 to 19·377) | (-2·372 to 111·583) | (3·219 to 54·278) | (-21·989 to 72·375) |
| East of England | 53·528 | 12·607* | 5·664* | 52·352 | 13·548*** | 39·598 | 22·587 | 17·064 |
|  | (-3·499 to 110·555) | (0·946 to 24·267) | (0·298 to 11·030) | (-4·585 to 109·290) | (5·794 to 21·303) | (-14·262 to 93·458) | (-2·369 to 47·543) | (-27·131 to 61·259) |
| London | 42·074 | 14·091* | 7·122* | 41·287 | 11·935** | 29·963 | 14·851 | 13·410 |
|  | (-18·723 to 102·872) | (1·644 to 26·539) | (1·247 to 12·996) | (-19·436 to 102·009) | (3·752 to 20·119) | (-27·302 to 87·228) | (-9·868 to 39·570) | (-34·735 to 61·554) |
| South East | 36·050 | 11·148 | 6·046* | 34·422 | 7·900* | 27·249 | 14·653 | 10·695 |
|  | (-20·592 to 92·691) | (-0·298 to 22·594) | (0·776 to 11·316) | (-22·132 to 90·975) | (0·363 to 15·436) | (-26·399 to 80·898) | (-9·678 to 38·984) | (-33·830 to 55·220) |
| South West | 17·327 | 0·421 | 1·770 | 17·465 | 3·133 | 15·620 | 30·847* | -13·309 |
|  | (-51·576 to 86·230) | (-14·871 to 15·712) | (-5·281 to 8·821) | (-51·498 to 86·428) | (-7·438 to 13·705) | (-49·854 to 81·094) | (1·823 to 59·871) | (-66·600 to 39·982) |
| **Referral Year (ref=2019)** |  |  |  |  |  |  |  |  |
| 2020 | -120·358*** | -7·324*** | 2·328 | -119·919*** | -13·277*** | -106·385*** | -51·330*** | -55·025** |
|  | (-166·857 to -73·858) | (-10·767 to -3·880) | (-0·588 to 5·244) | (-166·409 to -73·429) | (-20·678 to -5·875) | (-150·426 to -62·344) | (-74·478 to -28·181) | (-89·895 to -20·155) |
| 2021 | 80·174*** | 5·956** | 17·632*** | 81·385*** | 25·504*** | 55·536* | 3·409 | 41·458* |
|  | (35·728 to 124·620) | (2·195 to 9·718) | (14·534 to 20·730) | (36·948 to 125·821) | (18·523 to 32·486) | (12·847 to 98·226) | (-17·321 to 24·139) | (6·452 to 76·464) |
| 2022 | 161·694*** | -4·230 | 14·439*** | 164·044*** | 33·827*** | 129·609*** | 34·673** | 81·657*** |
|  | (109·373 to 214·015) | (-10·695 to 2·235) | (9·622 to 19·257) | (111·738 to 216·351) | (25·553 to 42·101) | (79·878 to 179·341) | (10·824 to 58·522) | (41·451 to 121·862) |
| **Referral Month (ref=Jan)** |  |  |  |  |  |  |  |  |
| Feb | -48·513** | -11·786*** | -6·877** | -48·137** | -0·980 | -47·296** | -17·478* | -30·413* |
|  | (-80·763 to -16·262) | (-17·842 to -5·730) | (-11·011 to -2·743) | (-80·341 to -15·934) | (-6·667 to 4·707) | (-77·652 to -16·941) | (-31·068 to -3·889) | (-55·218 to -5·608) |
| Mar | -51·303*** | -5·071* | -3·374* | -50·863*** | 0·232 | -51·126*** | -16·655* | -34·307** |
|  | (-81·197 to -21·410) | (-9·382 to -0·760) | (-6·458 to -0·290) | (-80·728 to -20·998) | (-5·235 to 5·698) | (-79·241 to -23·010) | (-29·887 to -3·423) | (-57·091 to -11·522) |
| Apr | -22·893 | -13·135*** | -7·961*** | -22·439 | 3·465 | -25·464 | 9·813 | -28·845 |
|  | (-63·285 to 17·498) | (-18·201 to -8·069) | (-11·719 to -4·203) | (-62·800 to 17·922) | (-3·705 to 10·634) | (-63·839 to 12·911) | (-7·449 to 27·075) | (-58·964 to 1·274) |
| May | -27·495 | -14·818*** | -6·938*** | -27·218 | 2·957 | -29·914 | -7·182 | -19·344 |
|  | (-64·191 to 9·200) | (-20·067 to -9·569) | (-10·526 to -3·349) | (-63·879 to 9·443) | (-4·466 to 10·379) | (-64·224 to 4·395) | (-22·978 to 8·613) | (-46·793 to 8·104) |
| Jun | -19·939 | -10·272*** | -3·363 | -19·771 | -1·375 | -18·326 | -17·738 | 0·248 |
|  | (-59·760 to 19·881) | (-15·103 to -5·441) | (-7·037 to 0·311) | (-59·580 to 20·039) | (-8·566 to 5·817) | (-56·232 to 19·580) | (-35·871 to 0·395) | (-29·830 to 30·325) |
| Jul | 53·916* | -2·257 | 3·621* | 54·328* | 7·412 | 46·483* | -8·083 | 51·680** |
|  | (10·720 to 97·111) | (-7·071 to 2·558) | (0·044 to 7·199) | (11·173 to 97·482) | (-0·331 to 15·154) | (6·483 to 86·483) | (-24·140 to 7·973) | (18·129 to 85·231) |
| Aug | 47·949* | -1·459 | 3·335 | 48·238* | 8·749* | 38·935* | 2·955 | 32·178* |
|  | (8·786 to 87·113) | (-6·378 to 3·459) | (-0·206 to 6·876) | (9·099 to 87·376) | (1·810 to 15·688) | (1·776 to 76·095) | (-13·714 to 19·625) | (1·069 to 63·287) |
| Sep | 71·745*** | -7·691** | -0·259 | 72·074*** | 7·211* | 64·421** | 9·534 | 49·164** |
|  | (30·799 to 112·691) | (-12·575 to -2·807) | (-4·046 to 3·528) | (31·147 to 113·001) | (0·482 to 13·940) | (25·094 to 103·748) | (-7·778 to 26·847) | (17·233 to 81·094) |
| Oct | 44·072 | 1·167 | 4·482* | 44·578 | 6·555 | 37·641 | 1·706 | 35·710* |
|  | (-14·331 to 102·475) | (-3·654 to 5·989) | (0·805 to 8·160) | (-13·801 to 102·958) | (-3·699 to 16·810) | (-13·620 to 88·902) | (-19·118 to 22·530) | (1·291 to 70·128) |
| Nov | 65·299*** | -0·465 | 4·652** | 66·175*** | 12·006*** | 53·776** | 3·426 | 44·994** |
|  | (30·027 to 100·570) | (-5·345 to 4·414) | (1·238 to 8·066) | (30·908 to 101·441) | (5·473 to 18·539) | (20·535 to 87·017) | (-11·408 to 18·260) | (17·278 to 72·710) |
| Dec | 82·172*** | 0·626 | 5·485* | 83·251*** | 21·086*** | 61·698*** | 19·044* | 34·851* |
|  | (43·645 to 120·698) | (-4·946 to 6·197) | (1·312 to 9·659) | (44·746 to 121·756) | (13·867 to 28·306) | (25·737 to 97·659) | (2·304 to 35·785) | (5·463 to 64·238) |
| Constant | -183·644*** | 97·718*** | 55·674*** | -183·673*** | -82·914*** | -105·088* | -93·453*** | -80·941* |
|  | (-281·572 to -85·716) | (83·011 to 112·425) | (46·631 to 64·717) | (-281·551 to -85·794) | (-98·837 to -66·991) | (-197·380 to -12·795) | (-146·382 to -40·523) | (-156·593 to -5·290) |

**Note:** N=1,471,142, 95% Confidence Intervals based on clustered standard errors at general practice (N=1,432) level in parentheses. * p<0·05, ** p<0·01, *** p<0·001.

**Supplementary Table 19: Coefficients from Models in Table 4 – healthcare cost – 1 year follow-up**

|  | (1) | (2) | (3) | (4) | (5) | (6) | (7) | (8) |
| --- | --- | --- | --- | --- | --- | --- | --- | --- |
|  | Primary Care and Hospital | All primary care | General Practitioner | All Hospital | Outpatient | Admitted Patient Care | Admitted Patient Care - Emergency | Admitted Patient Care - Elective |
| 2 year Lag of Outcome | 0·552*** | 0·413*** | 0·537*** | 0·552*** | 0·551*** | 0·551*** | 0·490*** | 0·575*** |
|  | (0·540 to 0·564) | (0·403 to 0·423) | (0·534 to 0·539) | (0·540 to 0·564) | (0·543 to 0·560) | (0·537 to 0·565) | (0·446 to 0·535) | (0·566 to 0·584) |
| Age | 6·351*** | -2·191*** | -1·184*** | 6·382*** | 2·277*** | 4·101*** | 4·900*** | 3·663*** |
|  | (4·282 to 8·420) | (-2·468 to -1·915) | (-1·351 to -1·016) | (4·313 to 8·450) | (1·915 to 2·639) | (2·113 to 6·089) | (2·468 to 7·333) | (1·904 to 5·422) |
| Age^2 | -0·044*** | 0·010*** | 0·008*** | -0·044*** | -0·022*** | -0·022* | -0·043*** | -0·020* |
|  | (-0·063 to -0·025) | (0·008 to 0·012) | (0·006 to 0·009) | (-0·062 to -0·025) | (-0·025 to -0·019) | (-0·040 to -0·003) | (-0·063 to -0·023) | (-0·037 to -0·003) |
| Female=1 | -67·191*** | 5·919*** | -5·242*** | -66·660*** | -4·134* | -63·091*** | -16·714** | -43·983*** |
|  | (-85·478 to -48·905) | (4·019 to 7·820) | (-6·223 to -4·261) | (-84·963 to -48·356) | (-7·287 to -0·982) | (-80·647 to -45·534) | (-26·748 to -6·679) | (-56·759 to -31·207) |
| **IMD Deciles - Income (Ref=1)** |  |  |  |  |  |  |  |  |
| 2 | 13·976 | -3·629 | -0·123 | 14·227 | 1·568 | 12·624 | 15·374* | -5·033 |
|  | (-20·674 to 48·625) | (-8·252 to 0·994) | (-2·715 to 2·470) | (-20·388 to 48·842) | (-5·546 to 8·683) | (-20·018 to 45·265) | (0·317 to 30·431) | (-32·798 to 22·731) |
| 3 | -5·154 | -6·786** | -1·091 | -4·793 | -2·561 | -2·233 | 13·655 | -18·708 |
|  | (-40·694 to 30·385) | (-11·526 to -2·045) | (-3·634 to 1·452) | (-40·336 to 30·749) | (-9·599 to 4·478) | (-35·352 to 30·887) | (-4·547 to 31·856) | (-46·313 to 8·896) |
| 4 | 1·752 | -4·467 | -0·113 | 2·083 | -1·975 | 4·130 | 7·252 | -7·173 |
|  | (-33·229 to 36·734) | (-9·278 to 0·344) | (-2·628 to 2·402) | (-32·918 to 37·084) | (-9·193 to 5·243) | (-28·671 to 36·931) | (-9·934 to 24·438) | (-34·791 to 20·445) |
| 5 | -3·142 | -3·165 | 0·051 | -2·970 | -1·308 | -1·588 | -6·723 | -4·152 |
|  | (-38·974 to 32·689) | (-8·382 to 2·053) | (-2·673 to 2·776) | (-38·824 to 32·883) | (-8·282 to 5·666) | (-34·962 to 31·787) | (-22·258 to 8·812) | (-31·667 to 23·363) |
| 6 | 11·332 | -6·174* | -0·183 | 11·825 | 1·768 | 10·116 | 13·584 | -11·616 |
|  | (-23·264 to 45·927) | (-12·196 to -0·152) | (-3·095 to 2·729) | (-22·760 to 46·411) | (-5·328 to 8·864) | (-22·376 to 42·609) | (-2·838 to 30·005) | (-38·754 to 15·522) |
| 7 | 19·396 | -7·479** | -2·317 | 19·903 | 1·525 | 18·490 | 6·224 | 0·732 |
|  | (-16·121 to 54·913) | (-13·061 to -1·898) | (-5·181 to 0·548) | (-15·615 to 55·420) | (-5·871 to 8·921) | (-14·504 to 51·485) | (-9·799 to 22·247) | (-26·976 to 28·439) |
| 8 | 7·028 | -7·267* | -1·851 | 7·530 | -1·291 | 8·843 | 8·567 | -13·847 |
|  | (-27·843 to 41·899) | (-12·997 to -1·537) | (-4·786 to 1·084) | (-27·364 to 42·423) | (-8·232 to 5·650) | (-23·842 to 41·528) | (-8·306 to 25·439) | (-41·240 to 13·546) |
| 9 | 13·090 | -5·538 | -1·249 | 13·494 | -1·360 | 14·883 | 4·041 | -6·344 |
|  | (-22·918 to 49·097) | (-12·137 to 1·060) | (-4·268 to 1·769) | (-22·509 to 49·498) | (-8·486 to 5·765) | (-18·551 to 48·317) | (-14·937 to 23·020) | (-33·685 to 20·996) |
| 10 = Least | -8·311 | -5·972 | -2·611 | -8·001 | -2·925 | -5·047 | -5·698 | -21·081 |
|  | (-43·914 to 27·293) | (-13·304 to 1·360) | (-5·718 to 0·496) | (-43·651 to 27·648) | (-10·644 to 4·794) | (-38·266 to 28·172) | (-26·290 to 14·893) | (-48·957 to 6·796) |
| **Ethnicity (ref White)** |  |  |  |  |  |  |  |  |
| Asian | 5·334 | 3·006 | -3·400* | 5·914 | -6·485* | 12·009 | 0·038 | 6·271 |
|  | (-21·031 to 31·699) | (-5·570 to 11·583) | (-6·520 to -0·279) | (-20·523 to 32·351) | (-12·078 to -0·892) | (-12·588 to 36·606) | (-13·251 to 13·326) | (-13·870 to 26·412) |
| Black | 6·079 | 1·416 | 0·996 | 5·964 | -1·637 | 7·305 | 1·829 | 3·697 |
|  | (-31·564 to 43·723) | (-4·739 to 7·572) | (-2·558 to 4·550) | (-31·692 to 43·621) | (-9·506 to 6·231) | (-27·422 to 42·031) | (-17·726 to 21·383) | (-22·261 to 29·655) |
| Mixed/Multiple | -27·980 | 14·050*** | 5·734** | -28·331 | -8·805 | -19·791 | -29·552* | 13·621 |
|  | (-84·377 to 28·416) | (8·009 to 20·091) | (1·525 to 9·942) | (-84·693 to 28·031) | (-20·786 to 3·176) | (-71·919 to 32·337) | (-58·455 to -0·650) | (-22·590 to 49·832) |
| Other | 16·299 | 22·263*** | 10·686* | 14·943 | 8·999 | 5·480 | 37·405 | -39·671 |
|  | (-103·997 to 136·594) | (9·944 to 34·582) | (1·102 to 20·269) | (-105·358 to 135·244) | (-18·908 to 36·906) | (-108·023 to 118·984) | (-12·147 to 86·958) | (-151·072 to 71·730) |
| unknown | 38·917 | 12·979** | 8·975*** | 37·696 | 11·480 | 25·994 | -7·300 | 23·828 |
|  | (-19·228 to 97·063) | (4·756 to 21·202) | (4·597 to 13·353) | (-20·322 to 95·714) | (-0·022 to 22·982) | (-29·977 to 81·965) | (-26·177 to 11·578) | (-28·009 to 75·665) |
| **Number of LTCs (ref =0)** |  |  |  |  |  |  |  |  |
| 1 | 4·240 | 18·391*** | 4·529*** | 4·949 | 0·272 | 4·893 | 5·631 | 1·162 |
|  | (-11·825 to 20·304) | (15·912 to 20·870) | (3·071 to 5·987) | (-11·092 to 20·989) | (-2·890 to 3·434) | (-10·109 to 19·896) | (-4·198 to 15·459) | (-9·266 to 11·589) |
| 2 | 16·010 | 20·018*** | 1·811* | 17·876 | 1·173 | 17·244 | 14·227* | 9·234 |
|  | (-3·177 to 35·197) | (16·405 to 23·631) | (0·155 to 3·468) | (-1·244 to 36·996) | (-2·264 to 4·610) | (-0·157 to 34·645) | (0·793 to 27·661) | (-2·412 to 20·880) |
| 3 | -10·776 | 18·751*** | -0·720 | -7·554 | -3·524 | -3·059 | 9·917 | -4·382 |
|  | (-35·022 to 13·470) | (14·022 to 23·480) | (-2·573 to 1·132) | (-31·674 to 16·566) | (-8·245 to 1·198) | (-25·093 to 18·974) | (-9·515 to 29·349) | (-18·141 to 9·376) |
| 4 or more | -21·963 | 13·139*** | -2·622** | -14·619 | -18·879*** | 6·990 | 12·236 | -8·417 |
|  | (-64·203 to 20·277) | (6·142 to 20·135) | (-4·449 to -0·795) | (-56·519 to 27·281) | (-24·729 to -13·029) | (-32·227 to 46·206) | (-18·704 to 43·176) | (-26·113 to 9·279) |
| **Region (Ref=North East)** |  |  |  |  |  |  |  |  |
| North West | 6·741 | 39·044*** | 11·224** | 4·272 | -16·700 | 21·325 | 24·044 | -3·497 |
|  | (-83·509 to 96·992) | (15·819 to 62·270) | (2·838 to 19·609) | (-86·001 to 94·545) | (-33·557 to 0·158) | (-59·760 to 102·411) | (-19·414 to 67·503) | (-56·466 to 49·472) |
| Yorkshire and Humberside | -24·100 | 52·661*** | 8·118** | -29·199 | -15·098* | -13·559 | 15·738 | -22·870 |
|  | (-95·685 to 47·484) | (32·658 to 72·665) | (2·099 to 14·138) | (-100·521 to 42·124) | (-26·591 to -3·605) | (-81·466 to 54·347) | (-27·136 to 58·612) | (-65·367 to 19·627) |
| East Midlands | 85·993* | -1·968 | 2·213 | 85·449* | 18·816** | 66·866 | 33·815 | 33·941 |
|  | (11·041 to 160·946) | (-25·006 to 21·070) | (-4·264 to 8·690) | (10·762 to 160·135) | (6·840 to 30·792) | (-7·428 to 141·160) | (-11·715 to 79·346) | (-14·719 to 82·600) |
| West Midlands | 71·691* | 16·948 | 3·322 | 69·305 | -0·474 | 70·039* | 36·760 | 32·941 |
|  | (2·055 to 141·327) | (-3·664 to 37·561) | (-2·440 to 9·083) | (-0·027 to 138·638) | (-10·970 to 10·022) | (3·143 to 136·936) | (-3·296 to 76·817) | (-10·115 to 75·997) |
| East of England | 63·735 | 21·633* | 5·151 | 61·796 | 4·820 | 57·227 | 36·452 | 21·264 |
|  | (-4·288 to 131·759) | (1·629 to 41·637) | (-0·721 to 11·023) | (-5·901 to 129·493) | (-5·483 to 15·124) | (-8·241 to 122·696) | (-3·024 to 75·928) | (-20·529 to 63·058) |
| London | 35·555 | 19·218 | 3·031 | 34·647 | 2·174 | 32·580 | 16·155 | 14·695 |
|  | (-34·450 to 105·561) | (-1·685 to 40·121) | (-3·173 to 9·236) | (-35·026 to 104·321) | (-8·643 to 12·992) | (-34·447 to 99·608) | (-22·562 to 54·871) | (-29·443 to 58·833) |
| South East | 35·120 | 20·115* | 5·459 | 32·872 | 0·333 | 32·651 | 20·339 | 10·357 |
|  | (-32·386 to 102·626) | (0·312 to 39·919) | (-0·272 to 11·189) | (-34·322 to 100·066) | (-10·050 to 10·716) | (-32·573 to 97·875) | (-18·302 to 58·979) | (-31·768 to 52·481) |
| South West | 49·039 | 1·194 | 0·346 | 49·256 | -7·834 | 57·279 | 45·378* | 14·197 |
|  | (-28·705 to 126·783) | (-24·335 to 26·723) | (-6·785 to 7·476) | (-28·297 to 126·808) | (-21·970 to 6·302) | (-16·347 to 130·906) | (3·065 to 87·692) | (-37·183 to 65·577) |
| **Referral Year (ref=2019)** |  |  |  |  |  |  |  |  |
| 2020 | -56·243* | -18·271*** | 2·176 | -54·796* | -9·001 | -45·471 | -12·996 | -31·808 |
|  | (-110·316 to -2·169) | (-22·478 to -14·065) | (-1·045 to 5·398) | (-108·858 to -0·734) | (-18·031 to 0·029) | (-96·748 to 5·806) | (-47·187 to 21·195) | (-64·550 to 0·934) |
| 2021 | 15·479 | -15·673*** | 17·801*** | 18·301 | 20·135*** | -1·913 | -13·667 | -0·013 |
|  | (-33·293 to 64·252) | (-20·315 to -11·031) | (14·385 to 21·217) | (-30·436 to 67·037) | (11·747 to 28·523) | (-48·862 to 45·037) | (-41·009 to 13·676) | (-31·771 to 31·744) |
| 2022 | 268·430*** | -25·192*** | 22·058*** | 272·586*** | 70·417*** | 201·919*** | 103·555*** | 83·407*** |
|  | (211·345 to 325·516) | (-32·383 to -18·001) | (17·491 to 26·625) | (215·526 to 329·647) | (60·457 to 80·376) | (147·784 to 256·054) | (74·143 to 132·967) | (45·772 to 121·043) |
| **Referral Month (ref=Jan)** |  |  |  |  |  |  |  |  |
| Feb | -11·650 | -13·291*** | -5·779*** | -11·254 | 1·271 | -12·585 | -7·783 | -5·550 |
|  | (-46·840 to 23·539) | (-19·742 to -6·839) | (-9·178 to -2·380) | (-46·422 to 23·913) | (-4·999 to 7·541) | (-44·970 to 19·800) | (-22·749 to 7·184) | (-31·519 to 20·420) |
| Mar | -25·102 | -7·035** | -3·986** | -24·896 | -1·694 | -23·279 | -9·656 | -13·602 |
|  | (-57·027 to 6·823) | (-11·816 to -2·253) | (-6·942 to -1·031) | (-56·788 to 6·996) | (-7·664 to 4·275) | (-52·518 to 5·961) | (-23·966 to 4·654) | (-36·607 to 9·404) |
| Apr | 103·139*** | -9·167** | -2·913 | 103·511*** | 19·491*** | 84·167*** | 67·455*** | 23·965 |
|  | (58·680 to 147·597) | (-14·768 to -3·565) | (-6·592 to 0·766) | (59·085 to 147·936) | (11·462 to 27·519) | (42·501 to 125·832) | (44·633 to 90·277) | (-7·354 to 55·284) |
| May | 85·941*** | -5·793 | 1·982 | 86·318*** | 28·237*** | 58·127** | 53·998*** | 7·865 |
|  | (45·631 to 126·250) | (-11·803 to 0·217) | (-1·525 to 5·490) | (45·990 to 126·646) | (20·136 to 36·339) | (20·668 to 95·585) | (35·934 to 72·062) | (-21·781 to 37·511) |
| Jun | 95·592*** | 0·305 | 5·597** | 96·007*** | 27·230*** | 68·801*** | 52·054*** | 17·712 |
|  | (54·949 to 136·234) | (-5·146 to 5·755) | (2·081 to 9·113) | (55·365 to 136·649) | (19·534 to 34·925) | (30·885 to 106·716) | (33·102 to 71·006) | (-13·021 to 48·444) |
| Jul | 155·817*** | 1·624 | 7·826*** | 156·350*** | 32·460*** | 123·914*** | 52·066*** | 68·860*** |
|  | (112·729 to 198·905) | (-3·661 to 6·908) | (4·559 to 11·093) | (113·284 to 199·416) | (22·274 to 42·645) | (85·005 to 162·823) | (33·829 to 70·302) | (37·088 to 100·631) |
| Aug | 122·396*** | 0·285 | 6·582*** | 123·071*** | 31·440*** | 91·518*** | 46·140*** | 41·077* |
|  | (78·421 to 166·371) | (-5·239 to 5·809) | (3·292 to 9·872) | (79·109 to 167·033) | (23·698 to 39·181) | (50·509 to 132·526) | (27·471 to 64·808) | (7·601 to 74·552) |
| Sep | 158·414*** | -3·624 | 5·763** | 159·245*** | 34·246*** | 124·878*** | 62·253*** | 56·241*** |
|  | (116·675 to 200·153) | (-8·805 to 1·558) | (2·224 to 9·302) | (117·499 to 200·992) | (26·995 to 41·496) | (85·797 to 163·960) | (44·048 to 80·459) | (24·824 to 87·658) |
| Oct | 82·233** | 0·449 | 7·753*** | 83·082** | 25·351*** | 57·818* | 37·073** | 20·690 |
|  | (22·025 to 142·442) | (-4·850 to 5·748) | (4·491 to 11·015) | (22·776 to 143·387) | (13·399 to 37·304) | (6·412 to 109·225) | (13·772 to 60·374) | (-12·033 to 53·414) |
| Nov | 108·710*** | -6·096* | 7·090*** | 109·895*** | 30·317*** | 79·424*** | 33·891*** | 39·410** |
|  | (67·384 to 150·035) | (-11·071 to -1·122) | (3·978 to 10·202) | (68·555 to 151·236) | (22·402 to 38·232) | (42·040 to 116·808) | (15·034 to 52·748) | (10·331 to 68·488) |
| Dec | 145·181*** | -7·415* | 6·615*** | 146·457*** | 38·219*** | 108·103*** | 48·429*** | 50·932** |
|  | (102·401 to 187·961) | (-13·231 to -1·599) | (2·891 to 10·340) | (103·707 to 189·208) | (30·364 to 46·074) | (69·008 to 147·199) | (29·765 to 67·092) | (20·411 to 81·453) |
| Constant | -307·224*** | 118·823*** | 42·586*** | -309·434*** | -87·776*** | -221·514*** | -167·225*** | -128·530*** |
|  | (-421·328 to -193·121) | (96·773 to 140·873) | (33·682 to 51·490) | (-423·395 to -195·473) | (-105·837 to -69·715) | (-328·825 to -114·202) | (-257·062 to -77·389) | (-201·471 to -55·589) |

**Note:** N=1,471,142, 95% Confidence Intervals based on clustered standard errors at general practice (N=1,432) level in parentheses. * p<0·05, ** p<0·01, *** p<0·001.

**Supplementary Table 20: Regression results for main coefficient of interest *referrals to social prescribing* on outcomes – healthcare use and cost for 6 months and 1 year follow-up on sub-sample from the stricter matching criteria**

|  | **(1)** | **(2)** | **(3)** | **(4)** | **(5)** | **(6)** | **(7)** | **(8)** |
| --- | --- | --- | --- | --- | --- | --- | --- | --- |
|  | Primary Care and Hospital | All primary care | General Practitioner | All Hospital | Outpatient | Admitted Patient Care | Admitted Patient Care - Emergency | Admitted Patient Care - Elective |
| **Appointment** |  |  |  |  |  |  |  |  |
| *6 months post* | 0·721*** | 0·534*** | 0·191*** | 0·089*** | 0·079*** | 0·011 | -0·003 | 0·026*** |
|  | (0·602 to 0·840) | (0·435 to 0·634) | (0·145 to 0·236) | (0·040 to 0·139) | (0·037 to 0·121) | (-0·003 to 0·026) | (-0·015 to 0·010) | (0·022 to 0·031) |
|  |  |  |  |  |  |  |  |  |
| *1 year post* | 0·364*** | 0·103 | 0·023 | 0·013 | -0·000 | 0·015** | 0·006 | 0·009*** |
|  | (0·220 to 0·507) | (-0·010 to 0·216) | (-0·013 to 0·059) | (-0·022 to 0·048) | (-0·029 to 0·028) | (0·004 to 0·026) | (-0·003 to 0·015) | (0·005 to 0·013) |
|  |  |  |  |  |  |  |  |  |
| **Cost** |  |  |  |  |  |  |  |  |
| *6 months post* | 66·367*** | 3·161* | 2·003* | 65·732*** | 3·875* | 61·629*** | 7·706 | 52·743*** |
|  | (43·710 to 89·025) | (0·747 to 5·575) | (0·252 to 3·754) | (43·081 to 88·384) | (0·313 to 7·437) | (40·409 to 82·848) | (-2·555 to 17·966) | (36·338 to 69·149) |
|  |  |  |  |  |  |  |  |  |
| *1 year post* | 58·169*** | 4·175** | 0·924 | 57·927*** | 2·537 | 55·144*** | 10·741 | 44·125*** |
|  | (33·715 to 82·623) | (1·288 to 7·062) | (-0·673 to 2·521) | (33·465 to 82·390) | (-1·538 to 6·613) | (32·274 to 78·013) | (-4·691 to 26·173) | (29·004 to 59·247) |

**Note:** N=928,397, 95% Confidence Intervals based on clustered standard errors at general practice (N=1,432) level in parentheses. * p<0·05, ** p<0·01, *** p<0·001. Models include covariates: outcome 2 years prior, age, age^2, female, ethnicity group, IMD – Income Domain Decile, government office region, and month and year fixed effects of referral date. Models adjusted using weights from exact matching which includes matching on primary care appointments in one year pre-referral.

**Supplementary Table 21: Multilevel Modelling: Regression results for main coefficient from of interest *referrals to social prescribing* on outcomes – healthcare use and cost for 6 months and 1 year follow-up**

|  | (1) | (2) | (3) | (4) | (5) | (6) | (7) | (8) |
| --- | --- | --- | --- | --- | --- | --- | --- | --- |
|  | Primary Care and Hospital | All primary care | GP | All Hospital | Outpatient | APC | APC - Elective | APC - Emergency |
| **Appointment** |  |  |  |  |  |  |  |  |
| *6 months post* | 2·540*** | 2·247*** | 1·029*** | 0·285*** | 0·230*** | 0·058*** | 0·009 | 0·061*** |
|  | (2·427 to 2·653) | (2·151 to 2·343) | (0·985 to 1·073) | (0·237 to 0·332) | (0·186 to 0·274) | (0·046 to 0·071) | (-0·002 to 0·021) | (0·057 to 0·065) |
|  |  |  |  |  |  |  |  |  |
| *1 year post* | 0·523*** | 0·527*** | 0·009 | -0·022 | -0·025* | 0·005 | -0·002 | 0·006*** |
|  | (0·429 to 0·617) | (0·450 to 0·604) | (-0·019 to 0·038) | (-0·050 to 0·007) | (-0·049 to -0·001) | (-0·005 to 0·014) | (-0·010 to 0·006) | (0·002 to 0·009) |
|  |  |  |  |  |  |  |  |  |
| **Cost** |  |  |  |  |  |  |  |  |
| *6 months post* | 41·354*** | 7·167*** | 2·476*** | 39·415*** | 2·927 | 36·915*** | 3·397 | 33·939*** |
|  | (23·389 to 59·319) | (5·386 to 8·948) | (1·149 to 3·802) | (21·457 to 57·372) | (-0·071 to 5·926) | (19·977 to 53·853) | (-4·085 to 10·879) | (20·218 to 47·660) |
|  |  |  |  |  |  |  |  |  |
| *1 year post* | 27·602** | 8·952*** | 0·462 | 27·595** | -0·085 | 27·922** | 5·029 | 24·198*** |
|  | (9·750 to 45·453) | (6·678 to 11·227) | (-0·784 to 1·709) | (9·748 to 45·442) | (-3·373 to 3·203) | (11·251 to 44·593) | (-3·756 to 13·814) | (11·166 to 37·230) |

**Note:** N=1,479,157, 95% Confidence Intervals based on clustered standard errors at the general practice (N=1,432) level in parentheses. * p<0·05, ** p<0·01, *** p<0·001. Models adjusted using probability weights, note this is different to analytical weights in main results in Table 4. Results comparable.

**Supplementary Table 22: Negative Binomial Models: Regression results for main coefficient from of interest *referrals to social prescribing* on outcomes – healthcare use and cost for 6 months and 1 year follow-up**

|  | (1) | (2) | (3) | (4) | (5) | (6) | (7) | (8) |
| --- | --- | --- | --- | --- | --- | --- | --- | --- |
|  | Primary Care and Hospital | All primary care | GP | All Hospital | Outpatient | APC | APC - Elective | APC - Emergency |
| **Appointment** |  |  |  |  |  |  |  |  |
| *6 months post* | 0·284*** | 0·312*** | 0·269*** | - | 0·135*** | 0·218*** | 0·139*** | 0·353*** |
|  | (0·267 to 0·302) | (0·292 to 0·333) | (0·254 to 0·285) | - | (0·111 to 0·160) | (0·189 to 0·247) | (0·093 to 0·184) | (0·329 to 0·378) |
|  |  |  |  |  |  |  |  |  |
| *1 year post* | 0·019*** | 0·011 | 0·008 | - | -0·002 | 0·058*** | 0·018* | 0·105*** |
|  | (0·009 to 0·030) | (-0·001 to 0·023) | (-0·001 to 0·016) | - | (-0·012 to 0·007) | (0·043 to 0·073) | (0·001 to 0·036) | (0·087 to 0·123) |
|  |  |  |  |  |  |  |  |  |
| **Cost** |  |  |  |  |  |  |  |  |
| *6 months post* | 0.069*** | -0.007 | -0.000 | 0.057*** | - | 0.081*** | - | - |
|  | (0.051 to 0.087) | (-0.022 to 0.008) | (-0.016 to 0.015) | (0.038 to 0.076) | - | (0.056 to 0.107) | - | - |
|  |  |  |  |  |  |  |  |  |
| *1 year post* | 0.055*** | 0.005 | 0.008 | 0.054*** | - | 0.079*** | - | - |
|  | (0.041 to 0.068) | (-0.006 to 0.015) | (-0.002 to 0.018) | (0.040 to 0.068) | - | (0.061 to 0.097) | - | - |
|  |  |  |  |  |  |  |  |  |

**Note:** N=1,479,157, 95% Confidence Intervals based on clustered standard errors at the general practice (N=1,432) level in parentheses. * p<0·05, ** p<0·01, *** p<0·001. “–“ indicates model did not converge.

**Supplementary Figures**

Figure S1A: Sample creation of case-control cohort to request data and linkages from CPRD

Patients not referred to social prescribing service after 1 July 2019

(N=11,971,596)

**Control**

Remove one practice with over 17,000 referrals on one day

(N=25,308)

Patients referred to social prescribing service after 1 July 2019

(N= 403,307)

**Treatment**

Registrations removed where not active between 1 July 2019 and 31 March 2024 (N= 21,057,403) and those registered less than one year (N=404,175)

Total = 21,461,578

Removing patients aged less than 18 at the end of the study period

(N=12,400,211)

Patients aged less 18 at end of study period

(N=2,591,302)

Removing practices registered outside of England and list size of less than 1,000 patients

(N=14,991,513))

Practices registered outside of England (N=12,677) and list size less than 1,000 (N=5,352)

Total = 18,029

Records acceptance for research purpose

(N=49,585,087)

Keeping those only with active registrations between 1 July 2019 and 31 March 2024 and registered for at least less than one year

(N=15,009,542)

Records not acceptable for research purposes according to CPRD

(N=7,590,356)

Records not eligible for linkage to HES Ethnicity and small-area data

(N=13,113,967)

Eligible for linkage to HES Ethnicity and small-area data

(N=36,471,120)

Patients in September 2024 version of CPRD Aurum Database

(N=57,175,443)

Figure S1B: Sample creation of case-control cohort to request data and linkages from CPRD

One treatment to five controls based on:

Age (+/- 5 years) and Gender (exact), additionally, one control was selected from the same general practice and four selected from different practices (no replacement)

Patients referred to social prescribing service after 1 July 2019

(N= 403,307)

**Treatment**

Patients not referred to social prescribing service after 1 July 2019

(N=11,971,596)

**Control**

Patients referred to social prescribing service after 1 July 2019

(N= 325,033)

**Treatment**

Patients not referred to social prescribing service after 1 July 2019

(N= 1,359,480)

**Control**

Additionally, sample selection using the same as above, but including:

Year of death (exact) if died during study period

Year joined/left (exact) if joined or left practice during study period

Figure S2: Exact matching and cohort used for analysis

Patients not referred to social prescribing service after 1 July 2019

(N= 1,359,480)

**Control**

Patients referred to social prescribing service after 1 July 2019 to 31 March 2024

(N= 325,033)

**Treatment**

Patients not referred to social prescribing service after 1 July 2019

(N=1,302,443)

**Control**

Patients referred to social prescribing service after 1 July 2019 to 31 March 2024

(N= 168,699)

**Treatment**

Exact matching, based on:

Number of long-term conditions (up to 4 or more)

Anxiety/depression

Referral year

Referral month

Total number of primary care appointments 6 months pre-referral

The control group is assigned a randomised index date drawn from the same distribution of referral dates as the treatment group.

Removing patients referred after 1 April 2022 (N=149,136)

Patients referred to social prescribing service after 1 July 2019 to 31 March 2022

(N= 175,897)

**Treatment**

**Supplementary Figure S3: Distribution of referrals for untreated patients**

**Supplementary Code 1: Stata Syntax for Identifying Staff and locations from Cons Source and Job Category lookup files**

```
foreach staff in gp nurse HCA pharmacist PA physiotherapist paramedic phlebotomist podiatrist therapist AP   midwife counsellor other {
**Identifying location from CPRD identifiers**
import delimited "ConsSource.txt", clear
ren id conssourceid
gen keep=0
**face to face**
gen facetoface=.
foreach desc in "GP Surgery" "Urgent Appointment" "Nurse Practitioner Surgery" "Seen in Nurses Surgery" "Surgery consultation" "Urgent Surgery" "Nurse Practitioner" "Duty Doctor Urgent Appointment" "Main Surgery" "Same Day Appointment" "Nurse Minor Illness Clinic" "Emergency Doctor" "Seen in GPs surgery" "G.P. Morning Surgery" "Nurse Sexual Health Appointment" "Diabetic Clinic" "Clinic" "Nurse's Treatment Room Clinic" "Minor Operations Clinic" "Surgery Emergency" "Surgery Attendance" "Same Day Clinic" "Nurse Assessment Clinic" "Nurse Surgery" "Branch Surgery" "Baby Clinic" "Minor Ops Clinic" "Emergency Gp Surgery" "Face to face consultation" "G P Consultation" "Nurse Run Family Planning Clinic" "Weekday Surgery" "Treatment Room" "Consultation" "Practice Nurse Clinic" "Emergency Nurse Clinic" "Emergency consultation" "G.P.Surgery Urgent Consultation" "Surgery Clinic" "G.P. Evening Surgery" "Seen in Health Centre" "GP Practice" "Clinic Premises" "Extra Appointment" "Seen by Practice Nurse" "Practice Nurse" "G.P Surgery (Pm)" "Emergency Appointment" "Treatment Room (Nurse)" "P.Nurse Clinic" "Open Surgery" "Saturday Morning Surgery" "Clinic note" "Surgery or Clinic" "Unbooked Clinic" "Emergency Surgery" "Surgery" "Urgent consultation" "Attendance" "Clinic NHS" "Community health clinic" "G P Ordinary Surgery" "Community Clinic" "Same day appointment" "Seen in GP unit" "Seen in general practitioner unit" "Face to face consultation" "Routine consultation" {
  replace facetoface= 1 if description == "`desc'"
  replace keep = 1 if description == "`desc'"
}
gen homevisit=.
**Home Visit**
foreach desc in "Home Visit" "Residential Home" "Visit-Home" "Home Visit - In Surgery Hours" "Home visit note" "Home of Patient" "Nurse Visit" "Normal Home Visit (08:00 - 11:00)" "Nursing home visit note" "Nursing Home" "Acute visit" "Daytime Visits patients home" "Seen in own home" "visit" "Residential home visit note"  "Follow-up/routine visit" "Home" {
  replace homevisit= 1 if description == "`desc'"
  replace keep = 1 if description == "`desc'"
}
gen telephone=.
**Telephone appointment**
foreach desc in  "Telephone Consultation" "Telephone call from relative/carer" "Duty Telephone Appt" "Telephone Appt" "Telephone" "Telephone Call" "Telephone Surgery" "Telephone call to a patient" "Phone" "Nurse Practitioner Telephone Advice" "Telephone call from a patient" "Telephone Advice" "Telephone encounter" "Duty Doctor Telephone" "Telephone call to relative/carer"  {
    replace telephone = 1 if description == "`desc'"
    replace keep  = 1 if description == "`desc'"
}
**Triage**
gen triage=.
foreach desc in "Telephone triage encounter" "Telephone (Triage)" "Nurse Triage Consultation" "Nurse Triage" "Triage" "Triage By Phone" "Nurse telephone triage" "Nurse Triage Clinic" "Telephone Triage" "Telephone Triage By Doctor" "Nurse Surgery Triage"{
  replace triage = 1 if description == "`desc'"
 /// replace keep  = 1 if description == "`desc'"
}
**Online**
gen online=.
foreach desc in "Consultation via video conference" "accuRx Consultation" {
replace online =1 if description == "`desc'"
replace keep  = 1 if description == "`desc'"
}

keep if keep ==1
drop keep
sa "`staff'_codes.dta", replace

**Identifying location of appointment from SNOMED Codes**
use " medical", clear
gen keep=0
**face to face**
gen facetoface=.
foreach desc in "GP Surgery" "Urgent Appointment" "Nurse Practitioner Surgery" "Seen in Nurses Surgery" "Surgery consultation" "Urgent Surgery" "Nurse Practitioner" "Duty Doctor Urgent Appointment" "Main Surgery" "Same Day Appointment" "Nurse Minor Illness Clinic" "Emergency Doctor" "Seen in GPs surgery" "G.P. Morning Surgery" "Nurse Sexual Health Appointment" "Diabetic Clinic" "Clinic" "Nurse's Treatment Room Clinic" "Minor Operations Clinic" "Surgery Emergency" "Surgery Attendance" "Same Day Clinic" "Nurse Assessment Clinic" "Nurse Surgery" "Branch Surgery" "Baby Clinic" "Minor Ops Clinic" "Emergency Gp Surgery" "Face to face consultation" "G P Consultation" "Nurse Run Family Planning Clinic" "Weekday Surgery" "Treatment Room" "Consultation" "Practice Nurse Clinic" "Emergency Nurse Clinic" "Emergency consultation" "G.P.Surgery Urgent Consultation" "Surgery Clinic" "G.P. Evening Surgery" "Seen in Health Centre" "GP Practice" "Clinic Premises" "Extra Appointment" "Seen by Practice Nurse" "Practice Nurse" "G.P Surgery (Pm)" "Emergency Appointment" "Treatment Room (Nurse)" "P.Nurse Clinic" "Open Surgery" "Saturday Morning Surgery" "Clinic note" "Surgery or Clinic" "Unbooked Clinic" "Emergency Surgery" "Surgery" "Urgent consultation" "Attendance" "Clinic NHS" "Community health clinic" "G P Ordinary Surgery" "Community Clinic" "Same day appointment" "Seen in GP unit" "Seen in general practitioner unit" "Face to face consultation" "Routine consultation" "Weekend consultation at surgery" "Out of hours consultation at surgery" "Bank holiday surgery consultation" "Same day appointment" "Seen in GP unit" "Seen in general practitioner unit" "Face to face consultation" "Routine consultation" {
  replace facetoface= 1 if term  == "`desc'"
  replace keep = 1 if term  == "`desc'"
}
gen homevisit=.
**Home Visit**
foreach desc in "Home Visit" "Residential Home" "Visit-Home" "Home Visit - In Surgery Hours" "Home visit note" "Home of Patient" "Nurse Visit" "Normal Home Visit (08:00 - 11:00)" "Nursing home visit note" "Nursing Home" "Acute visit" "Daytime Visits patients home" "Seen in own home" "visit" "Residential home visit note"  "Follow-up/routine visit" "Home" {
  replace homevisit= 1 if term  == "`desc'"
  replace keep = 1 if term  == "`desc'"
}
gen telephone=.
**Telephone appointment**
foreach desc in  "Telephone Consultation" "Telephone call from relative/carer" "Duty Telephone Appt" "Telephone Appt" "Telephone" "Telephone Call" "Telephone Surgery" "Telephone call to a patient" "Phone" "Nurse Practitioner Telephone Advice" "Telephone call from a patient" "Telephone Advice" "Telephone encounter" "Duty Doctor Telephone" "Telephone call to relative/carer" "Telephone consultation" "Telephone call by physician to patient or for consultation" "Telemedicine consultation with patient" "Consultation by telephone" "Telepractice consultation" {
    replace telephone = 1 if  term  == "`desc'"
    replace keep  = 1 if term  == "`desc'"
}
**Triage**
gen triage=.
foreach desc in "Telephone triage encounter" "Telephone (Triage)" "Nurse Triage Consultation" "Nurse Triage" "Triage" "Triage By Phone" "Nurse telephone triage" "Nurse Triage Clinic" "Telephone Triage" "Telephone Triage By Doctor" "Nurse Surgery Triage"{
  replace triage = 1 if term  == "`desc'"
 /// replace keep  = 1 if description == "`desc'"  | term  == "`desc'"
}
**Online**
gen online=.
foreach desc in "Consultation via video conference" "accuRx Consultation" "Consultation via multimedia" "E-mail consultation" "Consultation via telemedicine web camera" "Consultation via Typetalk relay service" "Consultation via SMS text message"  "Consultation via video conference" "Remote consultation" "Remote verbal consultation" "eConsultation via online application" "Consultation via instant messaging (synchronous)" "Consultation via video consultation" {
replace online =1 if term  == "`desc'"
replace keep  = 1 if term  == "`desc'"
}
keep if keep ==1
drop keep
format medcodeid %20.0g
tostring medcodeid, gen(consmedcodeid) format(%20.0g)
sa "`staff'_codes_term.dta", replace

**Job Categories**
import delimited "JobCat.txt", clear
gen keep =.
**GPs**
gen gp=.
foreach desc in "Clinical Medical Officer" "Medical Student" "Trust Grade Doctor - SHO level" "Senior Clinical Medical Officer" "General Medical Practitioner" "Sessional GP" "Staff Grade" "Locum GP" "Salaried General Practitioner" "Senior House Officer" "Trust Grade Doctor - Career Grade level" "Deputising Doctor" "Associate Practitioner - General Practitioner" "Specialist Registrar" "House Officer - Pre Registration" "Trust Grade Doctor - House Officer level"  {
    replace gp = 1 if description == "`desc'"
    replace keep = 1 if description == "`desc'"
}
**Nurses**
gen nurse=.
foreach desc in "Community Practitioner" "Community Mental Health Nurse" "Community Learning Disabilities Nurse" "Health Visitor" "Community Nurse" "Associate Practitioner - Nurse" "Student Nurse - Adult Branch" "Student Occupational Health Nurse" "Specialist Nurse Practitioner" "Student Practice Nurse" "Student District Nurse" "Student Nurse - Mental Health Branch" "Nurse Consultant" "Nurse Manager" "Nursing Cadet" "Staff Nurse" "Sister/Charge Nurse" "Student Community Mental Health Nurse" "Enrolled Nurse" "Modern Matron" "Advanced Practitioner" {
    replace nurse = 1 if description == "`desc'"
    replace keep = 1 if description == "`desc'"
}
**Health Care Assistant**
gen HCA=.
foreach desc in "Health Care Support Worker" "Healthcare Assistant" {
    replace HCA = 1 if description == "`desc'"
    replace keep = 1 if description == "`desc'"
}
**Other Clinical**
gen otherclinical=.
foreach i in pharmacist  physiotherapist  PA paramedic  midwife counsellor phlebotomist podiatrist therapist AP  other SPLW   {
	gen `i'=.
}
* Create a list of unique categories
foreach category in "Pharmacist" "Physiotherapist" "Physician associate" "Paramedic" "Midwife" "Counsellor" "Phlebotomist" "Podiatrist" "Other therapist" "Other" "Social Prescribing Link Workers" "Advanced Practitioner" {
    * For each category, code the individual entries
    if "`category'" == "Pharmacist" {
        foreach desc in "Pharmacist" "Pre-reg Pharmacist" "Clinical Pharmacist" "Clinical Pharmacist Advanced Practitioner" {
	    replace otherclinical= 1 if description == "`desc'"
	    replace pharmacist = 1 if description == "`desc'"
            replace keep = 1 if description == "`desc'"
        }
    }
    if "`category'" == "Advanced Practitioner" {
        foreach desc in "Advanced Practitioner" /*"Chiropodist/Podiatrist Advanced Practitioner" "Clinical Pharmacist Advanced Practitioner" "Paramedic Advanced Practitioner" "Physiotherapist Advanced Practitioner"*/ {
	    replace otherclinical= 1 if description == "`desc'"
	    replace AP = 1 if description == "`desc'"
            replace keep = 1 if description == "`desc'"
        }
    }
    else if "`category'" == "Physiotherapist" {
        foreach desc in "Physiotherapist" "Student Physiotherapist" "Physiotherapist Specialist"  "Physiotherapist Consultant" "Physiotherapist Manager" "Physiotherapist Advanced Practitioner" "Physiotherapist Specialist Practitioner" "First Contact Physiotherapists" {
          replace otherclinical= 1 if description == "`desc'"
	    replace physiotherapist = 1 if description == "`desc'"
            replace keep = 1 if description == "`desc'"
        }
    }
    else if "`category'" == "Physician associate" {
        foreach desc in "Clinical Assistant" "Assistant GP" "Physician Assistant" "Physician Associates" {
            replace otherclinical= 1 if description == "`desc'"
	    replace PA = 1 if description == "`desc'"
            replace keep = 1 if description == "`desc'"
        }
    }
    else if "`category'" == "Paramedic" {
        foreach desc in "Paramedic Specialist Practitioner" "Paramedic Consultant" "Paramedic" "Paramedic Manager" "Paramedic Advanced Practitioner" {
            replace otherclinical= 1 if description == "`desc'"
	    replace paramedic = 1 if description == "`desc'"
            replace keep = 1 if description == "`desc'"
        }
    }
    else if "`category'" == "Midwife" {
        foreach desc in "Midwife - Sister/Charge Nurse" "Midwife" "Midwife - Specialist Practitioner" "Midwife – Consultant" "Student Midwife" "Midwife – Manager" {
      	    replace otherclinical= 1 if description == "`desc'"
	    replace midwife = 1 if description == "`desc'"
            replace keep = 1 if description == "`desc'"
        }
    }
    else if "`category'" == "Counsellor" {
        foreach desc in "Counsellor" {
	    replace otherclinical= 1 if description == "`desc'"
	    replace counsellor = 1 if description == "`desc'"
            replace keep = 1 if description == "`desc'"
        }
    }
    else if "`category'" == "Phlebotomist" {
        foreach desc in "Phlebotomist" {
	    replace otherclinical= 1 if description == "`desc'"
	    replace  phlebotomist = 1 if description == "`desc'"
            replace keep = 1 if description == "`desc'"
        }
    }
        else if "`category'" == "Social Prescribing Link Workers" {
        foreach desc in "Social Prescribing Link Workers" {
	    replace otherclinical= 1 if description == "`desc'"
	    replace  SPLW = 1 if description == "`desc'"
            replace keep = 1 if description == "`desc'"
        }
    }
    else if "`category'" == "Podiatrist" {
        foreach desc in "Chiropodist/Podiatrist" "Chiropodist/Podiatrist Manager" "Chiropodist/Podiatrist Consultant" "Chiropodist/Podiatrist Specialist Practitioner" {
       	    replace otherclinical= 1 if description == "`desc'"
	    replace podiatrist = 1 if description == "`desc'"
            replace keep = 1 if description == "`desc'"
        }
    }
    else if "`category'" == "Other therapist" {
        foreach desc in "Multi Therapist" "Psychotherapist" "Occupational Therapist" "Therapist" "Occupational Therapy Specialist" "Practitioner" "Art Therapist Specialist Practitioner" "Art Therapist Consultant" "Art Therapist" "Multi Therapist Specialist Practitioner" "Drama Therapist" "Student Occupational Therapist" "Occupational Therapist Consultant" "Occupational Therapist Manager" "Art Therapist Manager" "Assistant Psychotherapist" "Speech & Language Therapist"  {
       	    replace otherclinical= 1 if description == "`desc'"
	    replace therapist = 1 if description == "`desc'"
            replace keep = 1 if description == "`desc'"
        }
    }
    else if "`category'" == "Other" {
        foreach desc in "Dietitian" "Radiologist" {
     	    replace otherclinical= 1 if description == "`desc'"
	    replace other = 1 if description == "`desc'"
            replace keep = 1 if description == "`desc'"
        }
    }
}
**Admin Staff**
gen admin=.
foreach desc in "Clerical Worker" "Community Administrator" "Waiting List Clerk" "Medical Director" "Manager" "Technician - Admin & Clerical" "Clinical Director" "Consultant Healthcare Scientist" "Analyst" "Health Records Clerk" "Mental Health Act Administrator" "Researcher" "System Administrator" "Desktop Support Technician" "Ward Clerk" "Special salary scale in Public Health Medicine" "Desktop Support Administrator" "Dispenser" "Support, Time, Recovery Worker" "Dental Assistant Clinical Director" "System Worker" "Information Officer" "Student Technician" "Cytoscreener" "Other Community Health Service - Admin Clerk" "Network Administrator" "Complaints Investigator" "Chair" "Receptionist" "Clinical Team Manager" "Trainee Scientist" "Healthcare Science Assistant" "Secretary" "Helpdesk Technician" "Social services care manager (mental health)" "Social work team manager (adults)" "Medical Secretary" "Call Operator" "Finance Director" "Healthcare Science Associate" "Clinical Application Administrator" "Community Team Manager" "Ward Manager" "Specialist Healthcare Scientist" "Health Records Administrator" "Technician - Additional Clinical Services" "Waiting List Manager" "Social Services information manager" "Helpdesk Administrator" "Chief Executive" "Biomedical Scientist" "Social services senior management" "Appointments Clerk" "Audit Manager" "Board Level Director" "Home Care organiser" "Network Technician" "Availability Monitor" "Non Executive Director" "Director of Nursing" "Clinical Coder" "Medical Laboratory Assistant" "Porter" "Nursery manager" "Medical Records Clerk" "Medical Records Manager" "Caldicott Guardian" "Senior Administrator" "Director of Public Health" "Clinical Director - Medical" "Specialist Healthcare Science Practitioner" "Technician - Healthcare Scientists" "Technician - PS&T" "Other Executive Director" "Healthcare Science Practitioner" "Senior Manager" "Healthcare Scientist" "Outpatient Manager" {
  replace admin = 1 if description == "`desc'"
  replace keep = 1 if description == "`desc'"
}
keep if keep ==1
drop keep
keep if `staff' ==1
sa "`staff'_staffcodes", replace
```
